# “Food Village”: An Innovative Alternative Food Network Based on Human Scale Development Economic Model

**DOI:** 10.3390/foods11101447

**Published:** 2022-05-17

**Authors:** Giordano Stella, Biancamaria Torquati, Chiara Paffarini, Giorgia Giordani, Lucio Cecchini, Roberto Poletti

**Affiliations:** 1 Department of Agricultural, Food and Environmental Sciences, University of Perugia, Borgo XX Giugno 74, 06121 Perugia, Italy; stellagiordano@libero.it (G.S.); bianca.torquati@unipg.it (B.T.); giorgia.giordani@studenti.unipg.it (G.G.); luciocecchini89@gmail.com (L.C.); 2 Sereco-Biotest Studi e Ricerche Ambientali, Via C. Balbo 7, 06121 Perugia, Italy; r.poletti@serecobiotest.it

**Keywords:** alternative food networks (AFNs), human needs, food sovereignty, Civil Economy (CE), Economy for the Common Good (ECG)

## Abstract

Although the different alternative food networks (AFNs) have experienced increases worldwide for the last thirty years, they are still unable to provide an alternative capable of spreading on a large scale. They in fact remain niche experiments due to some limitations on their structure and governance. Thus, this study proposes and applies a design method to build a new sustainable food supply chain model capable of realizing a “jumping scale”. Based on the theoretical and value framework of the Civil Economy (CE), the Economy for the Common Good (ECG), and the Development on a Human Scale (H-SD), the proposed design model aims to satisfy the needs of all stakeholders in the supply chain. Max-Neef’s Needs Matrix and Design Thinking (DT) tools were used to develop the design model. Applying the design method to the food chain has allowed us to develop the concept of the “Food Village”, an innovative food supply network far from the current economic mechanisms and based on the community and eco-sustainability.

## 1. Introduction

Overall, the food produced could feed about 10 billion people globally. However, the food system fails to achieve a fair allocation of resources [1]. Nearly 1.3 billion tons of food (about one-third of total production) are wasted every year, and approximately 56% of waste occurs in industrialised countries. However, they use better agricultural, transport, and conservation techniques [2].

In 2019, almost 690 million people were undernourished worldwide [3]. At the same time, by 2025, the prevalence of global obesity is predicted to reach an estimated 257 million adults, showing a rapid increase from 202 million in 2016 [4].

The agro-industrial system has enormous environmental impacts in biodiversity reduction, land degradation, aquifers’ pollution, greenhouse gas emissions, and air pollution from the transport of foodstuffs, among others [5]. Moreover, the current food system has produced considerable social costs for small producers worldwide (product sales price lower than the production price, reduction in income, increased phenomena of failure, marginalisation, and loss of self-esteem, land abandonment, and migration to cities and other countries [6].

Previously, the global food problem was addressed, promoting agro-industry, free market, and the production methods of the so-called “Green Revolution” (use of pesticides, chemical fertilisers, mechanisation, genetically modified organisms, etc.) by the major international organisations [7].

The large-scale distribution represents in the industrialized world the way in which the agro-food system organizes the consumers’ purchase of food. It is highly concentrated spatially and structurally and it is characterized by high levels of production and by long-distance import and export [8]; moreover, economic globalization and relatively cheap energy influence it [9]. This causes several negative environmental and social externalities [10].

However, recently, a change towards greater sustainability attributed to greater consumer sensitivity has been occurring towards socio-environmental issues [11,12] and numerous activist associations’ actions have been implemented [13], such as those of the international association of producers “La Via Campesina” (LVC). LVC states that, to achieve food security, “Food Sovereignty”, which is defined as “the right of people to healthy and culturally appropriate food produced through ecologically sound and sustainable methods, and their right to define their food and agriculture systems. It puts the aspirations and needs of those who produce, distribute and consume food at the heart of food systems and policies rather than the demands of markets and corporations”, [14] must be promoted.

Food Sovereignty was analysed in terms of its history, meaning, and local experience application by several authors [15,16,17].

According to LVC (See http://www.viacampesina.org for background) (accessed on 1 October 2021), only through a change in the economic paradigm, capable of guaranteeing democratic, eco-sustainable exchange processes based on respect for others, can food security be achieved [14]. In this perspective, the direct participation of all stakeholders in the governance of the “common good” is central to an organized political level; this direct participation is also crucial to community processes emerging by an approach from the low earners.

Some authors have highlighted the importance of participatory democracy techniques for the construction of policies [18,19] and the role of food territorial planning [20,21] in building resilience, nourishing, and promoting rural development. These tools are important to facilitate territorial planning by the public decision makers and the destination of economic resources for local development.

In the last three decades, within the discussions on emerging new forms of food supply and distribution that involve different actors, first of all, producers, and consumers, the Alternative Food Networks (AFNs) represent those that are more spread and analysed [22].

AFNs are food supply chains based on a community mould. In social relationality, eco-sustainable agricultural practices, public health, and social equity are promoted [23]. However, although the current AFNs are experiments of the food system towards a new economic paradigm, the current AFNs have remained, over time, a limited phenomenon.

Some authors [24,25,26] have stated that AFNs do not make a change in scale and do not produce a transition in the agri-food system due to the strong structuring of the food system, their relatively recent birth, and their bottom-up approach, remaining mostly niche initiatives. They affirmed that a way to facilitate the move beyond the niche is to focus on the community-orientated schemes’ role.

Agreeing with these authors, we argue that the reason AFNs have remained a niche phenomenon is that they fail to satisfy the needs of all stakeholders.

At the same time, the large-scale distribution creates environmental, social, and economic negative externalities.

From this perspective, both AFNs and large-scale distribution can be considered inefficient economic processes.

The aim of this paper is to propose an innovative AFN that can satisfy stakeholders’ needs involved in the food chain.

Specifically, to create a new eco-sustainable agri-food chain based on collective well-being and social equity, which promotes food security and can be spread on a large scale, in our opinion, it is first required to identify an adequate theoretical economic framework.

Therefore, it became necessary to analyse the needs of all stakeholders involved in the food chain and the intimate connection between them. Aiming to design the economic processes, a theoretical framework based on the principles of Civil Economy (CE) [27,28], the Economy for the Common Good (ECG) [29,30], and the theory of Human Scale Development (H-SD) by Max-Neef [31] was adopted. All of these heterodox models promote a sustainable vision that is the base of both all AFNs and the new food network chain we want to propose.

Aiming to enlarge the scale of impact of AFNs, we referred in particular to Max-Neef’s [31] model that theorizes that the economy has to respond to all needs of stakeholders. 

All these considerations and methodological approaches were used to develop the “concept” of an innovative food network: “The Food Village”.

The paper is organized in the following manner. In the Section 1.1 subparagraph we present the concepts of AFNs and the relevant literature on the topic, while in the Section 1.2 we compare four AFNs examples. In Section 2 “Materials and Methods”, firstly, in Section 2.1 subparagraph, we describe the economic models underlying the new food supply chain model. In the Section 2.2 subparagraph, we present Max-Neef’s matrix for needs analysis, while in the Section 2.3 subparagraph we describe a design method to conceive the innovative AFN for a new economic paradigm through Max-Neef’s matrix. In the Section 2.4 subparagraph we describe the application of the proposed design process to develop the new food supply chain. In Section 3 the concept of the innovative food network that emerged from the designing process is discussed. A short discussion follows.

### 1.1. Alternative Food Networks: Past and Actuality

Following the first food scandals linked to large-scale distribution, and since the 1980s, there has been a radical change in consumer demand, the so-called “food turning-point” [32], that has caused greater importance to be attached to the transparency of the production processes [33]. Meanwhile, the first environmental movements, ambitious to overturn the modernisation paradigm in the food sector, also contributed to this change.

Considering this background, a new way of thinking about the supply chain has begun taking shape. The organic system was the substratum on which national and international networks of producers and consumers were created. However, farmers have begun revealing the economic unsustainability of the large-scale distribution system and agriculture productivity. The loss of power along the supply chain and large-scale production has been determined to crush small- and medium-sized farms [34].

By adopting more efficient production techniques and promoting high-quality products, the farmers redeemed their position within the agri-food system; this process, together with consumers’ contribution in search of more sustainable food supply chains, has promoted the development of AFNs [35]. Among several definitions, Maye and Kirwan [36] (p. 1) described AFNs as “organised flows of food products connecting people (consumers) who care about the moral aspect behind their consumption practices. These people meet those (producers) who want a fair price for the way they produce food, far from the dominant (or conventional) logic of the market”. Jarosz [37] (p. 1) affirmed that “alternative food networks represent efforts to re-spatialize and re-socialize food production, distribution and consumption in North America, Europe and Australia”.

Terms such as AFNs and “Short Food Supply Chains” (SFSCs) are often used indiscriminately in such a way that the reduction in commercial nodes is the main feature of AFNs [38]. Instead, the “localisation” is given by an assortment of factors, and it is reductive to stop at the spatial conception alone. If taken individually as a criterion for evaluating the location of a given supply chain, the geographical configuration varies from radically local to radically global chains, with an infinity of intermediate cases in between. Therefore, for an exhaustive localisation analysis, several factors such as the product’s identity (typicality, processing, tradition), management organisation of the supply chain, and technologies used [39] have to be considered.

From another perspective, Watts et al. [40] argued that AFNs are distinct from conventional supply chains based on their commitment and potential subordination to global chains (i.e., those supply chains that operate in a global neoliberal policy).

Another central aspect that outlines the boundary between conventional and alternative systems is the involvement of the consumer and the level of relationality established in the exchanges between the players of the supply chain [23]. Opitz et al. [41] stated that the interaction with producers is one reason that encourages consumers to choose AFNs.

Ilbery and Maye [42] affirmed that the boundary between conventional systems and AFNs is not clearly defined: neither operates completely autonomously and differently due to the economic motives pushing the alternative producers to operate in both systems. This is an increasingly widespread phenomenon of hybridisation and “conventionalisation” [43] within alternative supply chains [44,45]. “Conventionalisation” refers to the contamination of alternative supply chains, which take on some of the characteristics of conventional supply chains, from which they originally wanted to go away. This can also occur following the attempt of “conventional players” to expand their market by including some of the characteristics of alternative food supply chains.

Le Velly [46] recognised the AFNs’ promise of diversity that a different organisation of the supply chain components should distribute benefits among producers, consumers, regions, and the environment. The difference is the characteristic that triggers the specific rules’ definition interconnected with conventional rules. Therefore, Le Velly [46] proposed to address the question “from the perspective of the organisational innovation processes activated” (p. 9). These innovation processes could be implemented by adopting specific “alternative rules” that are new ways of relating between the producer and consumer as well as new methods of production, transport, and different contracts, among others. However, some rules adopted from conventional supply chain models are not excluded, such as the infrastructure and knowledge of wholesalers. Similar to others, Le Velly [46] also regarded AFNs as ongoing, both emergent and making, rather than already shaped systemic entities.

Even if the AFNs represent a concrete proposal for transition, the discussion regarding both the maximisation of the potential of these initiatives to spread their social, ecological, and economic innovations to transform food systems, and avoiding the erosion of their authenticity is open and animate [22,25,26].

Indeed, Rossi [47] asserts that AFNs’ experiences are at a crucial point in their existence, also due to their growing interest in the demand side as well as the production; in fact, these experiences “on the one hand are both consolidating around their elements of alterity to the conventional food chains and, on the other, they are facing the challenge of growth and the interaction with the mainstream system” [22] (p. 4). Analysing five AFNs focused on community support agriculture, Rossi [47] argued whether the increase in AFNs could represent a way to enlarge the availability and affordability of the products by expanding the consumers’ access to these initiatives; equally there is the issue of conventionalisation.

In the next subsection, we will analyse four examples of AFNs in order to highlight their characteristics and to clarify their differences to understand the mechanisms that can facilitate or hinder the change in scale of AFNs.

### 1.2. A Comparison of Four Relevant AFNs

The four relevant AFNs analysed are: Italian Solidarity Purchase Groups (SPGs), the Organised Group of Supply and Demand (OGSD, GODO in Italian), the Community Supported Agriculture (CSA), and the Food Coop Park Slope (FCPS) model. Highlighting their potential and limitations has contributed to developing the “concept” of the new innovative food network proposed in this paper.

Born in Italy in the mid-1990s, the SPGs are a collective food supply practice including consumers who cooperate by buying food products or common goods directly from local producers at a fair price for both parties. The group participants first define a list of products that they collectively intend to purchase. Based on this list, the different persons compile orders collected to define a group order, transmitted to the producer (almost always organic). Finally, goods delivered are divided among the group members, and each one pays for his share [48].

The OGSD is a particular SPG where producers and consumers are associated with the Italian Association of Organic Agriculture (AIAB in Italian) to encourage matching the demand and supply of local organic products. It promotes responsible consumption based on seasonality, producer visibility, and product exchange without intermediation [49]. The OGSD facilitates purchases from member farms, manages product deliveries, provides information on the organoleptic and nutritional qualities of products, and promotes visits to member farms and training on organic farming [49].

The CSA is a community that is committed to supporting agricultural activities by sharing the risks and benefits of production with the farmer. The community co-designs the production and purchases a share of the production before each growing season. Hence, the farmer receives working capital in advance, thus obtaining greater financial security and better prices. Depending on their contribution, in return, the members receive regular farm products throughout the season [50].

The FCPS model is inspired by one of the oldest consumer food cooperatives in the United States, born in 1973 in New York. Its goal is to be a purchasing agent for its members, the only ones who can shop food and household items in the store. To have the possibility to buy into the selling point, all of them contribute with 2 h and 45 min of work every four weeks to the Food Coop. The FCPS focuses on sustainability and prefers selling environmentally sustainable products. Usually, the mark-up is only 21% compared to the wholesale price (26–100% in large-scale distribution) (See https://www.grubstreet.com/2018/04/history-of-the-park-slope-food-coop.html) (accessed on 10 November 2021). Additionally, the members’ work covers about 75% of the marketing costs associated with selling point employees (See http://foodcoop.film/) (accessed on 25 November 2021). Therefore, the Food Coop can be competitive and offer higher-quality products compared to large-scale distribution at lower prices. This model has also spread to Europe, where numerous cooperative supermarkets inspired by the FCPS experience have sprung up [51]. It has yielded significant results in creating social aggregation, a sense of community and solidarity, and promoting a fair and environmentally sustainable food supply.

As shown before, the literature on AFNs is vast and over time several authors have focused on different aspects. Recently, at the international level, there has been an open and growing debate on the social assumptions and on the characteristics of the economic processes necessary for the structuring, affirmation, and change in scale of the AFNs [52,53,54], among others.

Mount [52] wondered about the effects of the change in scale on the structure of AFNs, how this could affect the values that characterize them and the effectiveness with which they are able to translate them into coherent economic processes.

Wald, Hill [53] affirmed that reflecting on the scale concept helps both to perceive the development and the spread of food systems, and how certain alternative food system models could realize a “jumping scale”.

Using a multi-actor perspective framework, Poças Ribeiro et al. [54] explored the limiting and facilitating factors impacting the emergence and consolidation of different types of AFNs in three different countries. They underlined the fundamental role of organizers concerning the development of AFNs and that, at the same time, a wide scope of actions by governmental and non-governmental stakeholders supporting the development of more AFNs are needed.

We considered the characteristics of the four AFNs in order to highlight both the elements that hinder their change in scale and the elements that could facilitate the construction of a new AFN model.

These four AFNs are characterised by being governed by a “sharing economy” system. Business models based on sharing can be very different. Still, social well-being issues and the positive effects on the sustainable use of goods are a priority for all. A comparison to highlight their features was made concerning five key components: (i) the localisation (or proximity); (ii) the consumer involvement degree; (iii) the reasons for joining; (iv) the effects on sustainability; (v) the limits (Table 1).

Localisation or proximity is a discriminating aspect between the different forms of AFNs. In SPGs, the proximity between producer and consumer is high, as they collaborate to enhance local production, favouring organic or sustainable ones. In the OGSD, localisation is important (up to 80% in some cases) but not exclusive; supplying exclusively organic products often also involves farms located throughout the country. For CSAs and Food Coops, the proximity between supply and demand is not determined. In both cases, producers and consumers may never come into direct contact, and the mutual trust is based on the sharing of some fundamental values, such as product quality and fair prices. Despite this, in CSAs, proximity between producer and consumer is strongly recommended and it is usually the norm; sometimes, consumers support the production through manual labour.

Consumer involvement in SPGs is a fundamental part of the group: they voluntarily manage orders, deliveries, and quality control, among others, and the relationship between producer and consumer is constant and without intermediaries. Instead, in the OGSD, the consumer–producer collaboration either does not occur or occurs partially: consumers decide to subscribe to a specific organised group and support the producers through a membership fee. Sometimes, consumers can provide voluntary help at the headquarters of the local OGSD quarter. The supply–demand interaction occurs mainly through web platforms: orders are placed periodically, and then consumers can collect their shopping at the designated logistics points.

In CSAs, the producer is economically supported by the consumer; usually, at the beginning of the production year, they meet to co-plan the productions, and sometimes, the consumers contribute to manual labour helping the producers.

In Food Coops, the consumers’ involvement is active and high because they have to work within the coop to be able to buy from the selling point; they also decide which products they should be selling and participate in assemblies with the directors’ board.

In the SPGs, OGSD, and CSAs, the quality of the product, attention to the environment, and ethical–moral values are priority aspects for the members [49,55]. Specifically, the trust in the producer within the SPGs is what distinguishes the relationships before the support. Instead, in OGSD, the trust and support to the producer are based on the membership fee that each member–consumer pays to become part of it. In Food Coops and, particularly in Park Slopes, the desire to eat quality food and the possibility of being able to decide the provenience of the food supply unite people.

We focused on nutrition and environmental impact regarding the effects of the AFNs on consumers. SPGs, OGSD, and CSA, promote the consumption of local and organic foods. In the FCPS, healthy eating is the reason that led to its creation due to the difficulty in finding good quality foods in New York; this is why an assembly decision-making system open to all members was set up so that all the goods represent the will of the consumers. The products are preferably, but not exclusively, local, organic, vegan, and non-GMO, among others.

Concerning the impact on the environment, a critical aspect of CSA is that the consumer often overestimates their needs during the advance order, leading to food waste. Additionally, selling non-local producers’ goods, the FCPS model has a bigger impact on the environment because it uses more long-range transport with respect to the other AFNs.

Regarding the limits, the SPGs satisfy a restricted number of families’ demands: generally, no more than 50 families per group based on the territoriality feature of the productions. Therefore, if the requests exceed this threshold, a spin-off or a new group is arranged. Moreover, it is characterized by reduced product variability.

In OGSD, consumers can access a more varied product portfolio but still lower than the large-scale distribution.

In CSAs, inconsistencies between the ordered product and the one received often occur both in quality and quantity.

In SPGs, OGSD, and CSAs, the temporal and logistic accessibility limits consumers because the products could be withdrawn only during limited times of the week.

In SPGs and CSAs, the possible high involvement from all members could discourage many from joining; in the FCPS model, the high degree of involvement and work required could discourage many from joining.

From this analysis, the four AFNs could not make a change in scale and produce a transition in the agri-food system due to some constitutive limits. In particular, we have individuated some issues, including the failure to respond fully to consumer’s demand in terms of the assortment of food products, consumers requiring a medium–high involvement, the reduced time slots for accessibility to purchase, and uncompetitive prices.

It is necessary to develop an innovative model responding to all stakeholders involved in the supply chain, consumers, producers, and all operators, to surpass these critical issues and, thus, create a sustainable supply chain on a larger scale. Therefore, it is necessary to understand the economic principles and models that can form this innovative food chain structure. The next section reports the economic models that align with the principles and values underlying a new sustainable AFN.

## 2. Materials and Methods

### 2.1. The Economic Models Underlying the New Food Supply Chain Model

AFNs are characterised by human relatedness to distance themselves from the traditional supply chain model to accomplish the common good and environmental sensitivity.

To achieve these objectives on a large scale, we hypothesise that an innovative food supply chain based on an economic framework consistent with human values and ecology must be designed. Therefore, it seems necessary to extend Le Valley’s “promise of diversity” [46] to the epistemology of the economic model. Following this perspective, the “diversity” of the innovative supply chain model is based on the change in the economic paradigm. From this, the “diversity” of production, organisational, and governance systems can be ideated and designed in a coherent approach.

Therefore, three alternative economic models have been identified, together constituting the economic paradigm of the “Food Village”: the CE [27], ECG [29], and Economics of H-SD [31]. Starting from being different approaches, these models differ from the mainstream economic model because they focus on pursuing collective well-being and essential human needs. People are considered human beings rather than economic agents and are part of a single organism made up of the human community and the environment. From this perspective, the economy aims to achieve public happiness and to reach this result. Therefore, it must satisfy all essential human needs.

Born in Italy in the eighteenth century, the CE states that human beings act in terms of the market, friendship, reciprocity, gratuity, and fraternity in their economic actions. Hence, the market actor is seen as a person, not simply an individual, but a friendly economic agent. Public virtues replace the private interests of the political economy. In these terms, from Smith’s “invisible hand” that considered the market regulated based on the individual’s interests, it moves on to the “visible fabric” or to the civic virtues that each individual uses in the moment of exchange. Every economic operator should be endowed with moral qualities that can make a difference and enrich everyone. CE is based on five principles: (1) reciprocity, which makes the exchange personal and meaningful; (2) fraternity, which fosters diversity (cultural, religious, ethnic, etc.) and makes them compatible; (3) gratuity, open to others and treats them with respect, in reciprocity; (4) public happiness, arising from ethics and virtues as well as the common good and being the goal of society and the economy; (5) the plurality of economic actors, involving both public and private and profit and non-profit business, and overcoming the state–market duopoly, thereby making a more democratic economic system [27].

ECG is considered able to follow the necessary sustainable transformations on an economic, political, and social level across Europe (In 2016 an Opinion of the European Economic and Social Committee [56] was published concerning ECG where it was defined as a lever to the “transition towards a European Ethical Market which will foster social innovation, boost the employment rate, and benefit the environment”.) and worldwide. Even though the ECG is based on several disciplinary approaches derived from ethics, ecology, political science, social psychology, neurobiology, pedagogy, discussed over a long time (since 2010), ECG has been developed as a practical economic model. Therefore, it needs to be explored in the study of practices and developed clearly and in a structured way. Recently, Dolderer et al. [30], based on Felber [57], defined the “Common Good Economics” as “the science of satisfying the needs of the present and future human generations, in alignment with democratic values and ecological planetary boundaries” [30] (p. 7). The Common Good is well-thought-out in its highest and broadest significance [57], and the ability to achieve it results from economic success. Practically, the ECG has implemented the Common Good Balance Sheet (CGBS). It is a scorecard that measures how much public, private, or third sector activity contributes to the Common Good, based on the preservation of five fundamental values: human dignity, cooperation and solidarity (which count as one), ecological sustainability, social justice, and democratic co-determination and transparency [58]. The CGBS comprises 17 indicators that emerged from a matrix, intersected by the five fundamental values with the stakeholders of the analysed activity: suppliers, lenders, employees and holders, customers, partner companies, and the social and civil context, understood as territory, population, future generations, other human beings, and nature globally [59]. The CGBS is compiled directly to measure the contribution of the economic activity to the Common Good, verified and certified by external auditing: the more the activities are structured socially, ecologically, democratically, and jointly, the higher the score. Companies adopting the CGBS will be encouraged through tax exemption and easier access to public contracts and funds.

The Economics of H-SD states that the economic system has to respond to human needs. Unlike Maslow [60], Max-Neef [61] argued that it was not possible to affix a hierarchy to human needs and classified them as existential and axiological [62]. Existential needs are distinguished according to the dimensions of “being”, “having”, “doing”, and “interacting”. In contrast, the axiological needs are distinguished in subsistence, protection, affection, knowledge, participation, creativity, identity, freedom, and free time. Surpassing the existential and axiological needs, Max-Neef developed a matrix of needs. Through the matrix, it is possible to identify the satisfiers of human needs (Table 2), which represent ways of fulfilling individual needs.

For example, the need for knowledge can be satisfied through literature, a satisfier (way), while the good (means) used for this purpose is—potentially—a book. A satisfier could also be represented by interacting with new people and discovering new places, among others. Satisfiers are characterised by intangibility, as these represent how society approaches a need, while materiality characterises goods.

The H-SD Economy departs from the ideology behind the current economic model, where needs are met through material goods and services, seen more as an end than a means. Instead, the introduction of the satisfiers uses a less materialistic approach. Unlike needs, which remain the same for Max-Neef even between different historical periods and cultures, the satisfiers are influenced and modified by several factors. These are the organisational structure of a certain society, political system, and social practices. Moreover, they vary for each individual according to subjective attitudes, such as the character or the ethical and moral values. Ultimately, the relationship between human needs, satisfiers, and material goods is concretised in the Max-Neef matrix.

The Max-Neef matrix is an integral part of the design method that we propose in this study and, therefore, will be more explored in the next subparagraph.

### 2.2. Max-Neef’s Matrix for Needs Analysis

Max-Neef’s matrix promotes H-SD, a notion based on satisfying basic human needs and increasing self-confidence levels. With this perspective, it is possible to construct optimal synergies between the human being and environment, man and technologies, global processes and local activities, personal interests and social interests, participatory planning and private initiative, and civil society and the state [31].

The economic paradigm of H-SD raises the quality of life through a holistic understanding of people’s needs. Thus, each need is fulfilled by different sets of satisfiers that include all things that contribute to the satisfaction and well-being of the individual or the collective. In particular, the satisfiers corresponding to the existential needs of “being” refer to the individual or collective attributes, expressed with nouns, which refer to aptitudes—or particular inclinations—expressions of character, and personal values; the satisfiers corresponding to the needs of “having” represent the tools to contribute to these attributes. These tools can be norms, relational mechanisms, attitudes, or information. The satisfiers of “doing” are actions, individual or collective, expressed as verbs. These actions are aligned with the satisfiers of “being” and “having”. Finally, regarding the satisfiers corresponding to the need for “interacting”, places are used, in the spatial and temporal sense, in which people relate to and self-determine. Their articulation of the satisfiers is an essential element for realising total well-being, both individual and collective. If even one component of the matrix fails, the quality of life would inevitably be affected. Therefore, well-being arises at the level of the entire system once the right complementarities between the different dimensions are met: The well-being is not reduced to the accumulation of goods and services. The satisfiers are not exchanged and obtained through the market [63]; some have no exchange value and are neither exchanged nor exchangeable.

It should be remembered that the satisfiers do not respond univocally to needs. In the H-SD economic model, the needs are correlated, and a single satisfier can satisfy more than one. Conversely, a need may require more than one satisfier to be satisfied [31].

### 2.3. Designing Innovative AFN through the Max-Neef’s Matrix

Based on Max-Neef’s matrix, we defined a design method that we used to develop and to propose the innovative AFN “Food Village”, which could potentially respond to all stakeholder’s needs involved in the agri-food chain.

We based the design method starting from the Design Thinking (DT) approach. Today, DT represents a methodology of action that guides transformation, evolution, and innovation within the most varied economic sectors. DT was born to understand and identify spontaneous mental strategies of the designer, leading to the construction of a project [64].

Specifically, considering DT based on the designer’s ability to integrate human needs, the available material and technical resources, and a project’s constraints and opportunities, it is required that the designer is simultaneously analytical and emphatic, rational and emotional, methodical, and intuitive, oriented by plans and constraints, but spontaneous [65]. Some authors [66,67] call this attitude “abductive thinking”, from the idea of Peirce [68] that affirmed that the deduction or induction could not elaborate a new idea using data acquired in the past. In this sense, “abductive thinking” considers feelings and emotions as important as rationality. In DT, “abductive thinking” is related to the “perceptive cognition”, defined as “basic skill in the creation of new realities and artefacts” [64] (p. 3). In DT, “perceptual cognition” (feelings and emotions) and rationality are complementary to give full development to the “abductive reasoning”, that is, to thought with no anchorage regarding past data and experiences. According to Tschimmel [69], this premise is the basis of DT.

Stickdorn and Schneider [70] argued that the first step in developing a DT model is the design of the process itself; it changes according to the context in which the good or service is created. Therefore, it differs from project to project [64]. This vision corresponds to “constructivism”, the project’s success depending on the social actors and interaction environment of constructivism.

As mentioned before, we decided to base the construction of the design process on the Max-Neef matrix in order to identify the needs of stakeholders in the economic process.

Moreover, aiming to display the results of this work, DT techniques were considered; in particular, both the principles of abductive reasoning and storytelling, a communication and dissemination tool, were used. 

**Figure 1:**
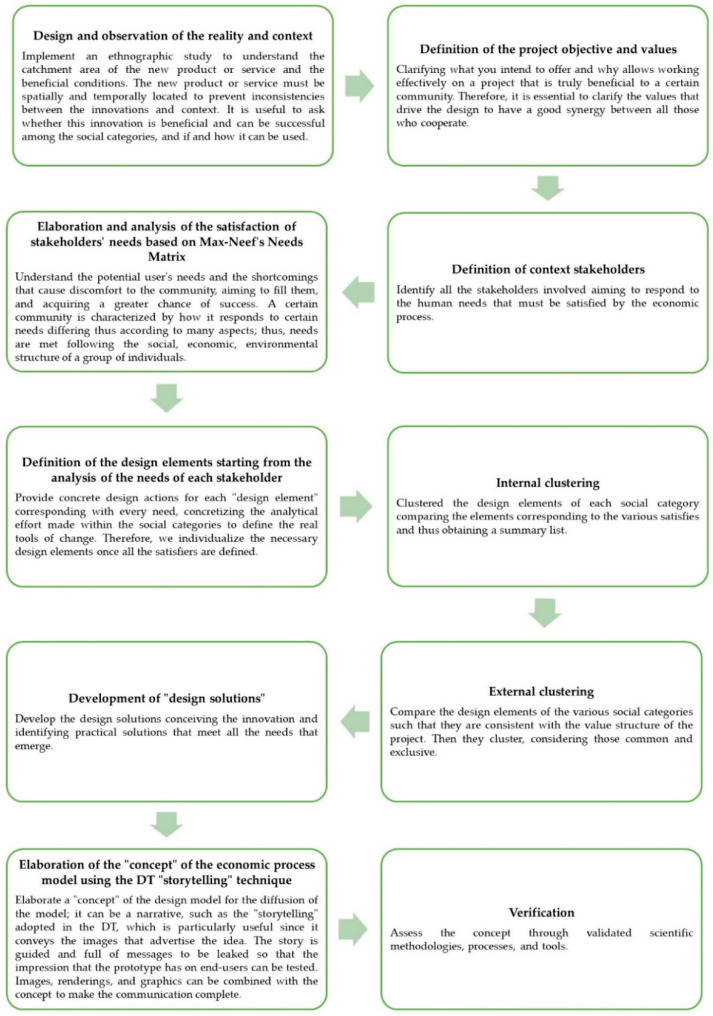
Design method.

The method arranged can be summarised in the following stages represented in Figure 1.

### 2.4. A New Food Supply Chain Model for a New Economic Paradigm

The method presented previously aimed to define rational modus operandi for developing the new supply chain model that respects social, economic, and environmental sustainability. The various phases and a brief explanation of each are reported below. Furthermore, the matrices of the needs of five social categories (consumers, producers, owners or financial partners, employees, collectivity) are designed. Supplementary Materials reports the matrix of consumers (Table S1), producers (Table S2), holders (Table S3), employees (Table S4), and collectivity (Table S5) and the clustered design elements (Table S6).

The application of the design method is described as follows:

**1. Definition and observation of the reality and context.** As previously presented, a literature review on AFNs was conducted, clarifying the difference between them to understand the mechanisms that can facilitate or hinder the change in scale within the value chain.

**2. Definition of the project objective and values.** The new supply chain model represents a virtuous example of social, economic, and environmental sustainability through good practices and a specific value heritage. The values of H-SD, CE, and the Common Good are shared, such as those of reciprocity, fraternity, gratuitousness, happiness, and human dignity, solidarity, and social justice, environmental sustainability, transparency, and co-determination.

**3. Definition of context stakeholders.** This definition was partly made by referring to a Manual for drafting a report on the Common Good Sheet (https://www.ecogood.org/) (accessed on 2 December 2021) and applied to the following five categories:

- Customers or consumers: the end-users of the goods or services provided by the supply chain.
- Producers or suppliers: subjects who sell their products through the supply chain channel.
- Holders or financial partners: those who make their own or third-party capital available. Financial service providers also belong to this category, companies that deal with transactions, insurance, and asset or financial advice.
- Employees or collaborators as active operators within the supply chain space: people who perform duties in the sales or purchase point, seasonal and non-seasonal agricultural workers, and all those who provide their service in one of the production process phases.
- Collectivity or social context, all groups that indirectly experience entrepreneurial actions while focusing on residents close to the supply chain and potentially critical NGOs.

**4. Elaboration and analysis of the satisfaction of stakeholders’ needs based on Max Neef’s Needs Matrix.** The needs of the five categories chosen were classified into existential and axiological [62]. Therefore, the five matrices report on the abscissa the need for being, having, doing, interacting, and on the ordinate, subsistence, protection, affection, understanding, participation, leisure, creativity, identity, freedom, and spirituality. The latter was added as essential for achieving complete well-being: spirituality can be understood as how human beings experience transcendence or connection to a higher system or power [71]. This sense of connectedness may or may not have religious affiliations. According to Max-Neef [31], the satisfiers must be declined according to precise criteria and the existential and axiological needs they respond to. Hence, these can take different forms, such as an attribute, noun, verb, or condition. The satisfiers were also identified by considering the degree to which they improve or inhibit individuals’ well-being concerning themselves, the community, or the environment [61]. Finally, their complementarity was considered to achieve complete well-being. The satisfiers’ classification based on how each affects the different dimensions of well-being has made it possible to highlight how the freedom and autonomy of individuals can be significantly influenced by changing or shaping certain social and economic mechanisms. The satisfiers are reported in the first four columns of Tables S1–S5 in the Supplementary Materials.

**5. Definition of the design elements starting from the analysis of the needs of each stakeholder.** This phase represents the real innovative contribution to the Max-Neef method in studying the needs of a certain community. Once the satisfiers had been identified, they were matched with concrete actions or tangible tools for satisfying needs. These were named “design elements”, representing the true answers of the applied analysis system. The design elements can be found in the last column of Tables S1–S5 in the Supplementary Materials.

**6. Internal clustering.** The clustering work allows a clearer view of the design elements for each category, thus having an operational purpose. In this study, the design elements of each social category are grouped into seven macro-areas: “Values/principles”, “Governance/training model”, “Training”, “Spaces”, “Communication”, “Cooperation”, “Characterizing elements”. The clustered elements are shown in the first five columns of Table S6 in the Supplementary Materials.

**7. External clustering.** The comparison between the design elements of the various social categories ascertains their consistency concerning the subjects and the value heritage attributed to the supply chain. Furthermore, this allows us to understand the useful answers for several categories simultaneously and streamline the supply chain design. The overall design elements can be found in the sixth column of Table S6 in the Supplementary Materials.

**8. Development of “design solutions”.** Some answers were elaborated. With these, we give substance to the design and organicity and completeness of the project work. The answers clearly outline the actions necessary for the realisation of the supply chain. The set of design solutions determines the concept of the supply chain model elaborated here.

**9. Elaboration of the “concept” of the economic process model using the DT “storytelling” technique.** Through this research work, a narrative form has been provided to the design work; then, the elaborated supply chain model has been described in words. Finally, in the following paragraph, a detailed discussion has been reported.

**10. Verification of the design work.** In DT, the innovative product or service is tested by submitting it to a panel of potential end-users to estimate its usefulness. Analyses were carried out on the propensity of consumers regarding the “The Food Village” concept using scientifically validated econometric tools. The results of this analysis will be presented in a future contribution.

## 3. Results

### The “Food Villages”: An Innovative Food Network Concept Proposal

The proposed “Food Villages” model promotes food resilience, health, environment care and defence, social aggregation, relationality, enhancement, and cultural biodiversity promotion, agroecology, and economic processes for the common good (increase in employment, fair compensation and rights of workers, appropriate production and services, etc.).

The model aims to establish a “Food Community” where the needs of all stakeholders can be satisfied. It is a prototype of an agri-food chain based on the ecological, civil, common good, and happiness economy principles to achieve the common good. The heart of the project is the “Community Pact for Food”, a set of shared values and practices around food, its production, impact on the environment, the economy, and society.

The base innovation is established on the concept that consumers, local producers, and the Food Village’s employees can be involved in the same legal entity, which combines supply, processing, and marketing to create a fair and ecological supply chain. The economic process could be ecological and achieve efficiency, redistribution, and relationality, thus becoming a tool for the development of the common good. Therefore, the “Community Cooperative” has been identified as the legal subject of the model.

Within this economic space, the needs of consumers and small–micro local producers are met and compared. The agricultural producers must create a stable income, receive fair compensation, operate in good working conditions, and improve the efficiency of production; at the same time, consumers need to constantly buy healthy and sustainable products at a fair price, and optimise their use of time, live spaces of relationship, and increase their awareness and self-determination.

The possibility to adhere to the “Community Cooperative” will be open and each kind of member will pay a membership fee to become part of it.

The products of the member farms conferring to this “Community Cooperative” will be sold within the Food Market.

Specifically, the food products’ supply sold in the Food Market will include three levels:

(1) “ultra-local”, characterized by the supply of members of the Food Village, located up to a maximum of 50 km away from the Food Market; this share of products will represent at least 20% of the total offer. All the ultra-local food products within the Food Market will be produced according to agroecological criteria or conferred by farms that are progressively in transition to agroecology, thus facilitating the involvement of local farms that would otherwise have been excluded.

(2) “local”, characterized by the supply of non-associated farms located up to a maximum of 200 km away from the Food Market; this share of products will represent at least 50–60% of the total offer.

(3) “local extended” characterized by the supply of non-associated farms, located up to a maximum of 500 km away from the Food Market; this quota will represent at least 20–30% of the total offer and will concern all those products that cannot be produced in the local area. The products that cannot be cultivated in the area, such as coffee, tea, cocoa, etc. will also be sourced beyond 500 km away from the Food Market.

This supply structure was designed to increase the goods variability, according to the quality criteria expressed by the consumers.

Household economy products with specific eco-sustainability certifications would also be sold in the Food Market.

The Food Market will allow consumers to have constant access to various products purchased in bulk or using eco-sustainable packaging where this is not possible. For those interested in reducing the use of cars, the Cooperative will organise a shopping delivery system three times a week. For those interested or in need, but also to reduce the use of cars, the Cooperative will organise a shopping delivery system three times a week.

The Food Market was planned to allow consumers to have constant access to a wide variety of products, something that rarely happens in short-chain models such as SPG and OGSD.

A micro-transformation system and storage will be created through modules owned by the cooperative (See for example Self-Globe modular plants (https://www.selfglobe.com/) (accessed on 10 June 2021) to reinforce the cooperative member’s local farmers’ role in the food supply chain.

The micro transformation could involve different kinds of activities such as a mill, a pasta factory, cheese factory, oil mill, seed cleaning, slaughterhouse, fruit and vegetable processing and transformation, etc.

The transformation processes will be carried out by a dedicated staff of the Cooperative. It will allow small and micro local agricultural farms participating in the Cooperative to transform their production; in fact, usually, these farms are forced to sell to wholesalers and large-scale retailers because they do not produce adequate quantities, thus foregoing fair compensation to limited quantities of agricultural production.

The micro-transformation modules will allow producers to raise their net income per hectare conferred through an internal redistribution of the surplus achieved by processed products. Road transport is reduced when the processing and marketing sectors are located in a single place.

Beyond the products supplied by local producers, the Food Market will be supplied according to traditional methods, based on the quality criteria expressed by the consumers themselves. Although there will be particular attention on the eco-sustainability of the supply chains involved, the Food Market of the “Food Village” could also sell all the kinds of products that a traditional supermarket usually sells. Thus, the Food Market purchasing agents will also be able to source from both non-local producers and distributors.

Contiguously to the Food Market, spaces for participatory democracy are provided for social assemblies, co-planning of prices and production, and the participatory certification of the local production. All spaces will be built according to bio-ecological architecture for their autonomous energy requirements. Additionally, they will provide permanent training to local producers on agroecology, business management, production processes, agronomic best available technologies (BAT), crop accounts, and price formation. Moreover, consumers will be provided with courses on healthy lifestyles (balanced nutrition, physical activity/sports, self-awareness practices, facilitation and participation techniques, education in relationships, etc.); territorial, national, and international dissemination and enhancement of food cultures will also be organised. 

**Figure 2:**
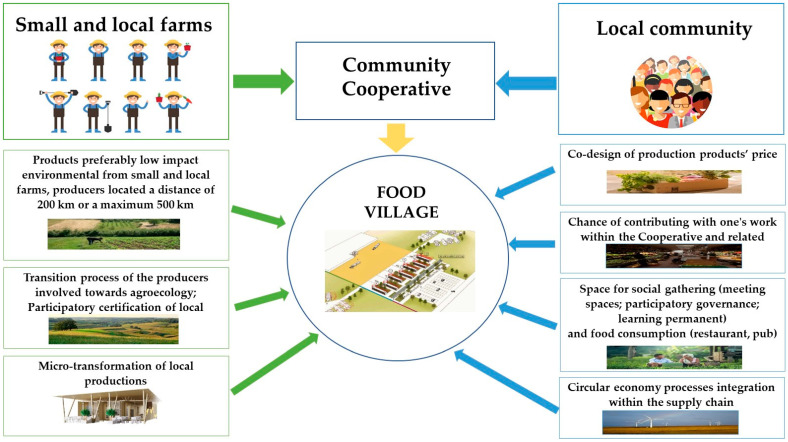
Food Village characteristics.

Figure 2 shows a brief graphical summary of the principal characteristics of the “Food Village”.

These spaces for participatory democracy could also be used to implement ecological transition projects (e.g., purchasing groups of green technologies such as solar and photovoltaic panels, electric bicycles, etc., as well as repairing and reusing objects).

Entertainment and catering sites are provided (such as a bar, restaurant, street food, theatre, etc.) to meet and attend artistic performances (music, presentations, books, readings, etc.) to facilitate the community aggregation. Restaurants mainly use products provided by members, thus creating another earning opportunity for the producers. Furthermore, the Cooperative could organise visits to the member farms to strengthen the sense of community, bond with the territory, and agricultural production.

“Food Village” is a replicable model; according to the needs and characteristics of a specific area, each “Food Village” Cooperative can open Food Markets separate from the headquarters to facilitate its increased usability. This is important in large cities where the space required to implement micro transformation and promote social aggregation and participatory democracy is unavailable in the city centres.

In each area, the Community Cooperative will aggregate the local offer and involve new farms based on the actual member’s consumption. This, along with the reduction in food waste at the trade phase, will stabilise the income for producers who could have the guarantee of selling their products even before production due to supply contracts. Furthermore, the procurement contracts established with the producer members could be confirmed annually, making the farm’s economic flows stable. A protocol could be defined within the cooperative regulations, governing the contractual relations between the cooperative and its producer members. The quality standards for production will be established for agroecological farmers and those in the agroecological transition process.

The product quality standards defined in the contracts will be verified through a participatory certification system in which the members are involved. If a producer fails to supply the Cooperative, the needs may be reallocated to other members in the same village or neighbouring villages. Software to coordinate operators and manage the compensation for production failures will be developed.

Moreover, the system will facilitate the work exchange and sharing of means of transport within the network to optimise resources, increase efficiency, and reduce costs of the production system. The Cooperative could facilitate the purchase, shared use of the production machinery, and reuse production waste within the farms involved or externally to implement circular productive and economic processes. This approach would raise the quality of the production system in ecological terms and also reduce production costs.

A “co-planning of production” model will be applied, stabilising producer members’ income and cost-saving by consumers in terms of a discount. This process will regard the “ultra-local” farmers and it will be developed in two phases. In the first phase, two months before the start of the agricultural season, consumers must indicate their weekly food needs (expressed in kg) for each food class (bread, pasta, vegetables, fruit, meat, etc.); namely, their food preferences in terms of the type of food consumed. Due to a dedicated calculation system, consumers will compare their food needs with an average balanced diet based on the Mediterranean diet. Thus, consumers could analyse their consumption and modify it if they deem it appropriate. Consequently, a pre-order to the cooperative will be placed based on the consumer’s food preferences using a matrix. Based on the previous year’s prices, the system estimates the expense and consumers decide whether to continue the order. Then, they must indicate the supply period: three, six months, or one year to simplify logistics for producers.

The second phase develops into participation paths to decide the prices of the products together. A “commission of members” (producers and consumers) will be created to define the annual price of food produced by farmer members. The definitive price from the co-design process will consider (1) the production costs and an agreed percentage surplus concerning the average national unitary income for the crop or food supplied; (2) the processing and marketing costs; (3) the replication or dissemination costs of the project (opening new Food Villages); (4) the discounts extended to members based on their degree of participation.

Once the prices are defined, the consumers can confirm, cancel, or modify their order. Once the order is confirmed, consumers will receive the products on a weekly basis, from the moment of harvest.

This process of price formation, excluding profits, will enable increased accessibility to food (fair price), adequately pay producers (fair compensation), and disseminate environmentally sustainable agricultural practices.

The “commission of members” supports the Food Market purchasing agents in selecting products that will be bought from producers outside the Cooperative to ensure that the product prices respect the principles of fairness and accessibility. Every three months, the commission will check if there is a need to revise selling prices.

The Food Market will be structured following the model of FCPS that requires each member to work three hours a month within it to buy products.

This model is now widely tested. It has yielded significant results both in creating social aggregation, a sense of community and solidarity, and in promoting a fair and environmentally sustainable way of supplying food. In fact, it is possible to buy high-quality food, often organic and local, at affordable prices within the FCPS. In the Food Villages, there will be voluntary (non-compulsory) possibilities to participate in the FCPS model.

Members making their contributions to the Food Village system can have access to a dedicated discount. Specifically, those who participate in the “co-planning of production” will access up to 10% of discounts concerning the co-planned products, while a 20% discount on all products sold in the Food Market will be provided for those who participate in the FCPS. Consumers that adhere to both models will access up to 30% of discounts concerning the co-planned products and a 20% discount on all products sold in the Food Market.

Members that participate in none of these activities, will be allowed to purchase and participate in all initiatives (educational activities, social events, etc.), while the consumers that are not members will be allowed only to purchase.

The “Food Villages” could represent “solidarity communities”, namely, social spaces where reciprocity is practised and one takes care of the other. Initiatives to meet the needs of the weaker social groups and emancipate those with difficulty in integration, including campaigns to satisfy fundamental human rights, promoting interculturality and inter-religiousness, work placement, right to housing, and food support (see the Last-Minute Market (https://www.lastminutemarket.it/) (accessed on 15 July 2021) experience and Banco Alimentare (https://www.bancoalimentare.it/it) (accessed on 15 July 2021), will be supported.

The community dimension and values expressed by the Food Villages and the technical, logistic, and governance models on which they are based on make Food Villages a real CE and ECG prototype. Therefore, to measure the impact of this model on the socio-economic and environmental fabric, the Food Villages will adhere to the guidelines of the ECG. Moreover, they will carry out the “Common Good Balance Sheet” annually. The “Common Good Balance Sheet” will also allow the Cooperative to foresee actions to improve Common Good and redirect the production processes of the partner farms of the Food Villages for the common good.

## 4. Discussion

This study proposed a new food supply chain model by reviewing four AFN models highlighting their characteristics, potential, and limits. Although AFNs promote an eco-sustainable paradigm change in the food chain, the current examples cannot provide an alternative capable of large scale spreading due to some constitutive limitations on their structure and governance explained previously. Therefore, we argue that an alternative should have the ability to “jump the scale”.

Our proposal is based on considering all the needs of all stakeholders in the supply chain simultaneously, as neither the current large-scale distribution nor the AFNs do that. Therefore, the Needs Matrix developed by Max-Neef within the economic model of H-SD was used to identify the needs (and their satisfiers) of all stakeholders in the supply chain. In contrast, the methodological framework of DT was used to develop a systematic and comprehensive design procedure. Therefore, the design model was built based on the criteria of the H-SD economic model and was intended to be a tool for spreading the vision and values, starting with the restructuring of economic processes.

Through the elaborate design method, the “Food Village” model was shaped and proposed in this study as a food supply network far from the current economic mechanisms and based on the community. Starting from the needs of the stakeholders, the design has allowed the strength of the community in creating economic processes that simultaneously allow sustainability, equity, reciprocity, and freedom as envisaged by the vision of the CE and the ECG to be enhanced. Basing governance on the community has favoured constructing a logistic and economic system capable of systemically incorporating all the system elements that favour sustainability (circular economic processes, agroecology, participatory governance, fair price or fair compensation, bio-architecture, etc.). Finalising the elements of the system to respond to current needs has opened up space for new processes within the economic exchange. An example is the co-design of prices. Establishing the Food Village on a community cooperative invests micro-transformation modules of local production on-site as a venture for all the stakeholders. Thus, this technology reduces the intermediary costs, leaving a greater margin for producers and consumers to compare and identify a fair price or fair compensation in a participatory process. Such a structured economic process promotes social interactions based on reciprocity by opening the space to a more cohesive society. Moreover, the model of the Food Village can be a powerful tool in promoting the rural economy, increasing the food resilience of the area, and reducing environmental impacts on the territory.

Following its application to the food supply chain, the proposed design system effectively identifies the satisfiers and design solutions capable of simultaneously promoting the different stakeholders’ satisfiers. Therefore, the Max-Neef Needs Matrix is an excellent analytical tool. Furthermore, the proposed method is useful and adaptable to economic contexts other than that of the Food Chain. Therefore, this procedure was considered a useful tool for the design of new economic processes capable of responding to the needs of all stakeholders.

The main limitation of this system is the length of time required for the need analysis process and a certain redundancy of the initial design elements. Therefore, in this model, these latest approaches are subsequently clustered.

This study aimed to provide a way of “moving alternative food networks beyond the niche” [25] (p. 1). Thus, further research and exchange of opinions are expected to arise from our food for thought.

## Figures and Tables

**Table 1:** Comparison between the AFNs.

		SPG	OGSD	CSA	FCPS
**Localisation/proximity**		**High**: It brings together local producers with consumers aiming to create a proximity exchange channel.	**Medium–High**: It is very important but not exclusive because OGSD supplies organic products exclusively.	**High**: Localisation is not an essential element but is strongly recommended.	**Medium**: The product’s prevalence comes from a distance of 200–500 km and other continents at the members’ wishes.
**Consumer involvement**		**High**: Consumers generally manage the organisational part of the group	**Medium–Low**: The products ordered are collected at distribution points or delivered home, so direct contact with the producers could be marginal.	**Medium–High**: Consumers support production before receiving products. Sometimes, they contribute to manual labour helping the producers.	**Very high**: Consumers work in it, decide which products should be selling and participate in assemblies by the directors’ board.
**Reasons for joining**		High product quality; ethical–moral values; trust in producers; social interactions; socio-political values; support to producers; ecological sustainability; fair price. Acquisition of new knowledge is not strictly a reason for joining.	High product quality; ethical–moral values; trust in producers; social interactions; socio-political values; support to producers; ecological sustainability; fair price. Acquisition of new knowledge is not strictly a reason for joining.	High products quality; ethical–moral values; trust in producers; socio-political values; support to producers; fair price; ecological sustainability; acquisition of new knowledge. Social interaction is not strictly a reason for joining.	High product quality; socio-political values; social interactions; support to producers; fair price; acquisition of new knowledge. Ethical–moral values, trust in producers, ecological sustainability are not strictly reasons for joining.
**Effects of sustainability**	Healthy Eating:	Consumers are very interested in healthy eating and nutrition education.	It promotes organic agriculture and the critical consumption of healthy foods.	It usually promotes organic agriculture.	It arises from the consumers’ need to find healthy foods in big cities.
	Use of natural resources:	Less impact of transport due to the high proximity supply–demand. Logistic system streamlined by governance. Low-input agriculture supported.	Less impact of transport due to the middle–high proximity supply–demand. Logistic system streamlined by governance. Organic agriculture supported.	Food waste can occur due to a wrong consumers’ estimate of the product they need in advance. Producers are often organic.	Imported products from other continents, if members request, could determine an environmental impact.
**Limits**		A limited number of families can join. Limited temporal and logistic accessibility. Reduced product variability. The possible high involvement from all members could discourage many from joining.	Limited temporal and logistic accessibility. Reduced product variability. A limited number of consumers supplied by local producers	Products ordered in advance may not be satisfactory at the time of delivery, both qualitatively and quantitatively. Limited temporal and logistic accessibility. The possible high involvement from all members could discourage many from joining. Food waste could occur.	The high involvement from all members could discourage many from joining. A consolidated system requests a large number of adherents willing to collaborate periodically over the years.

Source: Authors own elaboration.

**Table 2:** Matrix of Needs and Satisfiers.

	Being	Having	Doing	Interacting
**Subsistence**	Health, Adaptability, Sense of humour	Food, Shelter, Work	Feed, Procreate, Rest, Work	Social setting, Environment
**Protection**	Care, Equilibrium, Solidarity	Rights, Social security, Family	Cooperate, Plan, Help	Living space, Dwelling
**Affection**	Self-esteem, Respect, Passion	Friendship, Family, Relation with nature	Make love, Share, Cultivate, Appreciate	Privacy, Intimacy, Home, Togetherness
**Understanding**	Critical conscience, Curiosity, Discipline	Literature, Education, Teachers	Investigate meditate experiment	Groups, Community, Schools, Family
**Participation**	Dedication, Respect, Receptiveness	Rights, Responsibility duties, Work	Cooperate, Dissent, Agree on, Interact	Associations, Churches, Family
**Idleness**	Curiosity, Tranquillity, Imagination	Peace of mind, Games, Parties	Day-dream, Relax, Remember, Brood	Privacy, Intimacy, Free time, Landscape
**Creation**	Passion, Intuition, Imagination	Abilities, Skills, Method, Work	Work, Invent, Build, Compose, Design	Productive settings, Workshops, Time
**Identity**	Sense of belonging, Self-esteem	Language, Symbols, Religion, Values	Commit oneself, Grow, Recognise	Social rhythms, Maturation stages
**Freedom**	Autonomy, Boldness, Passion	Equal rights	Dissent, Choose, Disobey, Run risks	Temporal/spatial plasticity

Source: Max-Neef et al. [61] (p. 33).

## Data Availability

The data are available on request from the authors.

## Supplementary Materials

The following supporting information can be downloaded at: https://www.mdpi.com/article/10.3390/foods11101447/s1, Table S1: matrix of consumers, Table S2: matrix of producers, Table S3: matrix of holders, Table S4: matrix of employees, Table S5: matrix of collectivity, Table S6: the clustered design elements.
