# Supplementary material for: “Food Village”: An Innovative Alternative Food Network Based on Human Scale Development Economic Model
Source: Foods. 2022 May 17;11(10):1447. doi: 10.3390/foods11101447 (PMC9140569; doi:10.3390/foods11101447)
Supplement: Supplementary file 1 [file foods-11-01447-s001.zip › foods-1682105-supplementary.pdf]

"Food Village": An Innovative Alternative Food Network Based on Human Scale Development Economic Model

Table S1. Matrix of consumers

Customers' needs	Being	Having	Doing	Interacting	Design elements
**Subsistence**	- Physical health<br>- Sense of satisfaction in the purchase deed (Mental health)	- Access to adequate, balanced and healthy feed<br>- Healthiness and architectural accessibility of purchase/sale spaces<br>- Access to home economics goods<br>- Logistic and temporal accessibility proximity of purchase/sale spaces<br>- Variability of purchasable products<br>- Contact with natural elements	- Training on: human nutrition and production and transformation processes, bio-architecture and architectural accessibility, green spaces design<br>- Encouraging the eco sustainability of agri food production processes<br>- Requiring an optimal design in bio-architecture of the purchase/sale spaces and encourage an optimal green design of outdoor spaces<br>- Demanding the elimination of architectural barriers<br>- Demanding the opening of the shopping area at a maximum distance of 10 km and at least 5.5 days a week<br>- Identifying a minimum basket of food and household products	- Training spaces on: human nutrition and production and transformation processes, bio-architecture and accessibility, green design<br>- Participatory design of the purchase/sale spaces and of outdoor spaces (healthiness and accessibility of the spaces)<br>- Shopping space at a maximum distance of 10 km and at least 5.5 days a week<br>- Variated offered of food and household items portfolio.	- Courses and training spaces available on: human nutrition; production and transformation processes, bio-architecture and architectural accessibility green design<br>- Careful selection of products and suppliers, according to consumer preferences<br>- Participatory definition strategies for products present in the supply chain and participatory certification of products from local producers<br>- Co design programs for the spaces of the point of sale and purchase<br>- Home economics goods<br>- Food Market supermarket model (logistical and temporal accessibility, the variability of purchasable products)<br>- Strategies for involving customers in the co design of the external spaces of the point of sale and purchase
**Protection**	- Care and health<br>- Environmental care and regeneration<br>- Solidarity	- Access to adequate, balanced and healthy feed;<br>- Contact with natural elements<br>- Healthiness and architectural accessibility of purchase/sale spaces	- Training on: human nutrition and production and transformation processes, bio architecture; ecology and ecological indicators<br>- Demanding the eco sustainable approach of agri food production processes	- Training spaces on: human nutrition and production and transformation processes, bio architecture, ecology and ecological indicators<br>- Participatory governance that promotes the transparency of	- Courses and training spaces available to the customer on: human nutrition, production and transformation processes, bio-architecture and architectural accessibility,<br>- Ecology and ecological indicators

		– Minimization of the environmental impact of the purchase/sale spaces<br>– Minimization of the ecological footprint and the Carbon Footprint of production processes<br>– Territorial regeneration<br>– Minimization of food waste<br>– Collaboration and meetings between the stakeholders<br>– Social inclusion at work<br>– Economic accessibility to purchase	– Requiring an optimal design in bio-architecture of the purchase/sale spaces and encourage an optimal green design of outdoor spaces<br>– Participating in the definition of ecological production and marketing standards<br>– Promoting territorial regeneration projects<br>– Requiring procedures for minimizing food waste<br>– Promoting the creation of meeting spaces between stakeholders<br>– Requiring online shopping and home delivery methods<br>– Requiring social inclusion measures at work<br>– Requiring basic products with an affordable quality/price ratio to low income brackets	the production processes of the agri food chain<br>– Participatory design of the shopping areas (healthiness of environments) and green outdoor spaces<br>– Participatory definition of the ecological standards for production and commercialization<br>– Participatory design of territorial regeneration interventions<br>– Procedures for minimizing food waste<br>– Online spaces to order and to facilitate home delivery<br>– Participatory structure for identifying the fair prices of a basket of basic and essential products<br>– Spaces aimed at facilitating the construction of collaborative projects between stakeholders<br>– Social inclusion projects at work	– Careful selection of products and suppliers, according to customer preferences<br>– Strategies for the participatory definition of products present in the supply chain and participatory certification of products from local producers<br>– Bio-architecture building standards: natural construction materials, renewable energy sources, etc.<br>– Strategies for involving customers in the co design of the internal and external spaces of the point of sale and purchase<br>– Strategies for the participatory definition of ecological production and marketing standards, as well as for the reduction of food waste<br>– Participatory construction of territorial regeneration projects<br>– Online ordering and home delivery platform<br>– Participatory pricing strategies for a basket of basic and essential products<br>– Strategies to promote meetings and collaborative projects between stakeholders<br>– Participatory construction of projects for social inclusion at work
**Affection**	– Respect<br>– Relationality<br>– Solidarity	– Possibility to communicate with the managers of the purchase/sale area and to report problems and needs<br>– Social aggregation, training and culture	– Requiring spaces of communication with the managers of the purchase/sales area and spaces to report problems and needs<br>– Requiring spaces for social aggregation, training and culture fruition	– Spaces of communication with the managers of the purchase/sale area and reporting problems and needs<br>– Spaces for social gathering, training and cultural dissemination	– Presence of clear methods of communication with the managers of the purchase/sale area<br>– Presence of clear methods of reporting problems and needs<br>– Presence of refreshment points, pubs, a cultural centre and a park

		- Participatory governance of the supply chain<br>- Collaboration and meetings between the stakeholders<br>- Social inclusion at work<br>- Economic accessibility to purchase	- Requiring the opportunity to participate in the governance of the supply chain<br>- Promoting and activate collaborative projects between stakeholders<br>- Demanding social inclusion measures at work<br>- Promoting the creation of meeting spaces between stakeholders<br>- Requiring online shopping and home delivery methods<br>- Requiring social inclusion measures at work<br>- Requiring basic products with an affordable quality/price ratio to low income brackets	- Participatory structure of the governance of the supply chain<br>- Spaces aimed at facilitating the construction of collaborative projects between stakeholders<br>- Social inclusion projects at work<br>- Participatory structure for identifying the fair prices of a portfolio of basic and essential goods	- Definition of a participatory governance model of the supply chain<br>- Presence of spaces used for co design<br>- Participatory construction of projects for social inclusion at work<br>- Strategies for involving customers in the co design of the external spaces of the point of sale and purchase<br>- Strategies to promote meetings and collaborative projects between stakeholders<br>- Online ordering and home delivery platform<br>- Participatory pricing strategies for a portfolio of basic and essential goods
**Understanding**	- Critical consciousness<br>- Knowledge<br>- Curiosity	- Transparency of the point of purchase/sale governance<br>- Knowledge of producers and production and transformation processes<br>- Lifelong learning<br>- Transparency and comprehensibility of labels	- Requiring effective communication aimed at the transparency of the production chain<br>- Requiring and promote spaces of meeting with producers and transformation processors<br>- Requiring and promoting spaces for ongoing formation<br>- Training on healthy and environmentally friendly production and processing methods	- Co designed communication transparency process<br>- Spaces aimed at meeting with producers and transformation processors<br>- Training spaces on production techniques, transformation processes and healthy and environmentally friendly marketing methods<br>- Permanent participatory training space	- Communication transparency protocol of production chain<br>- Farms visits<br>- Training courses on production techniques, transformation processes and healthy and environmentally friendly marketing methods<br>- Definition of a model of ongoing participatory training

**Participation**	- Relationality<br>- Defense of one's rights and expression of one's personality<br>- Equity<br>- Sense of community	- Social aggregation, training and culture<br>- Participatory governance of the supply chain<br>- Collaboration and meetings between the stakeholders<br>- Social inclusion at work<br>- Possibility to communicate with the managers of the purchase/sale area and to report problems and needs<br>- Affordable access to quality food and household products<br>- Fair price, fair compensation<br>- Recognition of producers' needs and workers' rights<br>- Possibility of positively influencing the territorial ecosystem and social and relational system<br>- Possibility of positively influencing and supporting the local economy<br>- Possibility of positively influencing awareness and culture in one's own territory	- Requiring spaces for social aggregation, training and culture fruition<br>- Requiring the opportunity to participate in the governance of the supply chain<br>- Promoting and activate collaborative projects between stakeholders<br>- Requiring social inclusion measures at work<br>- Requiring spaces of communication with the managers of the purchase/sales area and spaces to report problems and needs<br>- Training on communication skills<br>- Training on participatory democracy models and good assembly practices<br>- Requiring basic products with an affordable quality/price ratio to low income brackets<br>- Requiring a participatory price training protocol<br>- Encouraging a participatory governance model through which to influence the social, economic and environmental processes associated with food<br>- Requiring and promoting spaces for ongoing formation	- Spaces for social gathering, training and cultural dissemination<br>- Participatory structure of the governance of the supply chain<br>- Spaces aimed at facilitating the construction of collaborative projects between stakeholders<br>- Social inclusion projects at work<br>- Spaces of communication with the managers of the purchase/sale area and of reporting problems and needs<br>- Spaces for training on: communication skills, participatory democracy and assembly techniques<br>- Participatory structure for identifying the fair prices of a portfolio of basic and essential goods<br>- Social inclusion projects at work	- Presence of refreshment points, pubs, a cultural centre and a park<br>- Definition of a participatory governance model of the supply chain<br>- Presence of spaces used for co designing processes<br>- Participatory construction of projects for social inclusion at work<br>- Strategies for involving customers in the co design of the external spaces of the point of sale and purchase<br>- Strategies to promote meetings and collaborative projects between stakeholders<br>- Presence of clear methods of communication with the managers of the purchase/sale area<br>- Presence of clear methods of reporting problems and needs<br>- Training courses on: participatory democracy assembly techniques and communication skills<br>- Participatory pricing strategies for a portfolio of basic and essential goods<br>- Multidimensional structure of participatory governance of the supply chain.
**Idleness**	- Relationality<br>- Carefreeness<br>- Fun<br>- Pleasure	- Social aggregation<br>- Lifelong learning<br>- Entertainment (Art and Culture)	- Requiring spaces for social gathering<br>- Requiring and promoting spaces for ongoing formation<br>- Requiring spaces for entertainment	- Spaces for social gathering, training and cultural dissemination<br>- Spaces for entertainment	- Presence of refreshment points, pubs, a cultural centre and a park<br>- Permanent training spaces (training courses, farm visits aiming to know the producers, training trips)<br>- Spaces used for entertainment

**Creation**	- Beauty<br>- Harmony<br>- Fantasy<br>- Collective well being	- Contact with natural elements<br>- Access to affordable purchase/sale and aesthetically pleasing outdoor spaces<br>- Entertainment (Art and Culture)<br>- Participatory governance of the supply chain, of training and cultural spaces	- Requiring a harmonious and aesthetically pleasing architectural design of the purchase/sale spaces and an optimal green design of outdoor spaces<br>- Requiring spaces for entertainment<br>- Requiring spaces for participatory democracy and co planning of educational and cultural activities	- Participatory design of the shopping areas (healthiness of environments) and green outdoor spaces<br>- Spaces for cultural events<br>- Participatory structure of the governance of the supply chain, of training and cultural spaces<br>- Spaces for participatory democracy sessions	- Strategies for involving customers in the co design of the internal and external spaces of the point of sale and purchase<br>- Pleasant architectural design of the purchase/sale spaces<br>- Presence of spaces for cultural events<br>- Definition of governance based on participatory democracy and the co planning of educational and cultural activities<br>- Spaces set up for participatory democracy sessions
**Identity**	- Self esteem<br>- Self affirmation<br>- Consistency with one's values<br>- Sense of belonging to the community and the territory	- Participatory governance of the supply chain, of training and cultural spaces<br>- Possibility to communicate with the managers of the purchase/sale area and to report problems and needs<br>- Access to local products<br>- Collaboration and meetings between the stakeholders<br>- Social aggregation<br>- Moments of historical re enactment of the food and wine of the area of interest.	- Requiring spaces for participatory democracy and co planning of educational and cultural activities<br>- Requiring spaces of communication with the managers of the purchase/sales area and spaces to report problems and needs<br>- Encouraging and contributing to the enhancement of local products<br>- Requiring spaces for collaboration and meetings between the stakeholders<br>- Requiring spaces for social aggregation<br>- Encouraging the training on the food and wine and cultural history of the territory.	- Participatory structure of the governance of the supply chain, of training and cultural spaces<br>- Spaces of communication with the managers of the purchase/sale area and reporting problems and needs<br>- Spaces aimed at facilitating the construction of collaborative projects between stakeholders<br>- Strategies to enhance local products<br>- Spaces for social gathering<br>- Spaces for training on the food and wine and cultural history of the area.	- Definition of governance based on participatory democracy and co planning of educational and cultural activities<br>- Presence of clear methods of communication with the managers of the purchase/sale area<br>- Presence of clear methods of reporting problems and needs<br>- Aggregation of the offer of local producers<br>- Facilitate strategies for collaboration between the stakeholders<br>- Presence of refreshment points, pubs, a cultural centre and a park<br>- Training courses on the food and wine history of the area.
**Freedom**	- Autonomy<br>- Choice<br>- Self esteem<br>- Self affirmation	- Participatory governance of the supply chain, of training and cultural spaces<br>- Possibility to communicate with the managers of the purchase/sale area and to report problems and needs	- Requiring spaces for participatory democracy and co planning of educational and cultural activities<br>- Requiring spaces of communication with the managers of the purchase/sales area and spaces to report problems and needs	- Participatory structure of the governance of the supply chain, of training and cultural spaces<br>- Spaces of communication with the managers of the purchase/sale area and reporting problems and needs	- Definition of governance based on participatory democracy and the co planning of educational and cultural activities<br>- Presence of clear methods of communication with the managers of the purchase/sale area

		- Possibility to develop and propose your own ideas around the design of spaces and governance of the supply chain	- Requiring spaces to share ideas on the design of spaces and governance of the supply chain at the meeting	- Spaces to share ideas on the design of spaces and governance of the supply chain at the meeting	- Presence of clear methods of reporting problems and needs
**Spirituality**	- Motivation<br>- Consistency with one's personal values<br>- Empathy towards others and nature<br>- Awareness and self knowledge	- Ethical and ecological governance of the supply chain, and according to universal human values<br>- Collaboration and meetings between the stakeholders<br>- Social aggregation<br>- Participatory governance of the supply chain, of training and cultural spaces<br>- Possibility to develop communication skills and empathy<br>- Possibility to acquire the awareness of own limits and potential<br>- Possibility to be present to oneself.	- Requiring ethical and ecological governance of the supply chain and verifying methods consistent with universal human values<br>- Requiring spaces for collaboration and meetings between the stakeholders, social aggregation, participatory democracy and co planning of educational and cultural activities<br>- Requiring training courses on communication skills and empathy, the practices of self knowledge.	- Governance of the supply chain built according to ethical and ecological principles and human values<br>- Participatory certification of the governance<br>- Spaces for collaboration and meetings between the stakeholders,<br>- social aggregation, participatory democracy and co planning of educational and cultural activities<br>- Training courses on communication skills and empathy<br>- Training courses on the practices of self knowledge and spaces used for this purpose.	- Definition of governance built according to ethical and ecological principles and human values<br>- and to human values<br>- Participatory certification of the governance (Common Good Balance Sheet, CSR, etc.)<br>- Strategies to facilitate collaboration between stakeholders<br>- Presence of refreshment points, pubs, a cultural centre and a park<br>- Definition of governance according to participatory democracy and the co planning of educational and cultural activities<br>- Training courses on communication skills and empathy, self awareness practices.

Table S2. Matrix of producers

Producers' needs	Being	Having	Doing	Interacting	Design elements
**Subsistence**	- Physical and mental health<br>- Security	- Stable contracts over time<br>- Fair productive requirements<br>- Fair income	- Obtain contract stability guarantees<br>- Obtain assurances on the fairness of the productions requested and the compensation paid	- Participatory definition of a guarantee protocol on the continuity of contracts between producers and owners, on the fairness of the requested productions and on the compensation paid	- Participatory definition between producers and owners of a guarantee protocol on the continuity of contracts, on the fairness of the productions requested and, on the compensation, paid
**Protection**	- Physical and mental health<br>- Security	- Stable contracts over time<br>- Fair productive requirements<br>- Fair income	- Obtain contract stability guarantees<br>- Obtain assurances on the fairness of the productions requested and the compensation paid	- Participatory definition of a guarantee protocol on the continuity of contracts between producers and owners, on the fairness of the requested productions and on the compensation paid	- Participatory definition between producers and owners of a guarantee protocol on the continuity of contracts, on the fairness of the productions required and on the compensation paid (Contracts protocol and Pact for food, co-production planning with owners and customers; participatory price formation)
**Affection**	- Respect<br>- Relationality<br>- Solidarity	- Possibility to communicate with the managers of the purchase/sale area and to report problems and needs<br>- Social aggregation, training and culture<br>- Participatory governance of the supply chain<br>- Collaboration and meetings between the stakeholders	- Requiring spaces of communication with the managers of the purchase/sales area and spaces to report problems and needs<br>- Requiring spaces for social aggregation, training and culture fruition<br>- Requiring the opportunity to participate in the governance of the supply chain<br>- Promoting and activate collaborative projects between stakeholders<br>- Promoting the creation of meeting spaces between stakeholders	- Spaces of communication with the managers of the purchase/sale area and reporting problems and needs<br>- Spaces for social gathering, training and cultural dissemination<br>- Participatory structure of the governance of the supply chain<br>- Spaces aimed at facilitating the construction of collaborative projects between stakeholders	- Clear methods of communication with those in charge of the buying/selling space<br>- Clear methods of reporting problems and needs<br>- Spaces aimed at the construction of collaborative projects: between producers, between producers and owners and between producer-clients (co-planning of production with owners and clients; participatory price formation; sharing of machinery between producers; sharing of workers between producers; coordination between producers for production failures; coordination between producers for disposal of production residues)

**Understanding**	- Critical consciousness<br>- Knowledge<br>- Curiosity	- Transparency of the point of purchase/sale governance<br>- Contractual clarity<br>- Lifelong learning	- Require transparency of point-of-purchase/sales governance processes<br>- Require clarity of contractual relationships<br>- Requiring and promoting spaces for ongoing formation	- Communication transparency protocol of the production chain<br>- Protocol of understanding on production and delivery methods<br>- Training spaces on production techniques and on healthy and eco-compatible transformation processes<br>- Spaces of permanent training	- Communication transparency protocol of the production chain<br>- Protocol of understanding on production and delivery methods (certifications, production failures, etc.)<br>- Training spaces on production techniques and on healthy and eco-compatible transformation processes<br>- Definition of a permanent participatory training model
**Participation**	- Relationality<br>- Defense of one's rights and expression of one's personality<br>- Equity<br>- Sense of community	- Social aggregation, training and culture<br>- Participatory governance of the supply chain<br>- Collaboration and meetings between the stakeholders<br>- Social inclusion at work<br>- Possibility to communicate with the managers of the purchase/sale area and to report problems and needs<br>- Stable contracts over time<br>- Fair productive requirements<br>- Fair income<br>- Recognition of producers' needs and workers' rights<br>- Possibility of positively influencing the territorial ecosystem and social and relational system<br>- Possibility of positively influencing and supporting the local economy<br>- Possibility of positively influencing awareness and	- Requiring spaces for social aggregation, training and culture fruition<br>- Requiring the opportunity to participate in the governance of the supply chain<br>- Promote the creation of spaces aimed at building collaborative projects: between producers, between producers and owners and between producers and clients<br>- Promoting and activate collaborative projects between stakeholders<br>- Requiring social inclusion measures at work<br>- Requiring spaces of communication with the managers of the purchase/sales area and spaces to report problems and needs<br>- Training on communication skills<br>- Training on participatory democracy models and good assembly practices<br>- Obtain contract stability guarantees<br>- Obtain assurances on the fairness of the productions requested and the compensation paid	- Spaces for social gathering, training and cultural dissemination<br>- Participatory structure of the governance of the supply chain<br>- Spaces aimed at facilitating the construction of collaborative projects between stakeholders<br>- Social inclusion projects at work<br>- Spaces of communication with the managers of the purchase/sale area and of reporting problems and needs<br>- Participatory definition of a guarantee protocol on the continuity of contracts between producers and owners, on the fairness of the requested productions and on the compensation paid<br>- Spaces for training on: communication skills, participatory democracy and assembly techniques<br>- Social inclusion projects at work	- Presence of refreshment points, pubs, a cultural centre and a park<br>- Definition of a participatory governance model of the supply chain<br>- Presence of spaces used for co designing processes<br>- Participatory construction of projects for social inclusion at work<br>- Strategies for involving producers in the co design of the external spaces of the point of sale and purchase<br>- Strategies to promote meetings and collaborative projects between stakeholders<br>- Presence of clear methods of communication with the managers of the purchase/sale area<br>- Presence of clear methods of reporting problems and needs<br>- Training courses on: participatory democracy assembly techniques and communication skills<br>- Participatory definition between producers and owners of a guarantee protocol on the continuity of contracts, on the fairness of the productions required and, on the compensation,

		culture in one's own territory	- Encouraging a participatory governance model through which to influence the social, economic and environmental processes associated with food<br>- Requiring and promoting spaces for ongoing formation		paid (Contracts protocol and Pact for food, co-production planning with owners and customers; participatory price formation)<br>- Multidimensional structure of participatory governance of the supply chain.
**Idleness**	- Relationality<br>- Carefreeness<br>- Fun<br>- Pleasure	- Social aggregation<br>- Lifelong learning<br>- Entertainment (Art and Culture)	- Requiring spaces for social gathering<br>- Requiring and promoting spaces for ongoing formation<br>- Requiring spaces for entertainment	- Spaces for social gathering, training and cultural dissemination<br>- Spaces for entertainment	- Presence of refreshment points, pubs, a cultural centre and a park<br>- Permanent training spaces (training courses, farm visits aiming to know the producers, training trips)<br>- Spaces used for entertainment
**Creation**	- Skills<br>- Professional skills<br>- Sensitivity<br>- Collective well being	- Possibility to find market for own productions and for new experimental products<br>- Participatory governance of the supply chain, of training and cultural spaces	- Obtain guarantees on the possibility of having sales space for experimental products<br>- Requiring spaces for participatory democracy and co planning of educational and cultural activities	- Sales spaces for experimental products<br>- Participatory structure of the governance of the supply chain, of training and cultural spaces<br>- Spaces for participatory democracy sessions	- Sales spaces for experimental products<br>- Definition of governance based on participatory democracy and the co planning of educational and cultural activities<br>- Spaces set up for participatory democracy sessions
**Identity**	- Self esteem<br>- Self affirmation<br>- Consistency with one's values<br>- Sense of belonging to the community and the territory	- Participatory governance of the supply chain, of training and cultural spaces<br>- Possibility to communicate with the managers of the purchase/sale area and to report problems and needs<br>- Access to local products<br>- Collaboration and meetings between the stakeholders<br>- Social aggregation<br>- Possibility to produce foods of the enogastronomic and cultural history of the territory	- Requiring spaces for participatory democracy and co planning of educational and cultural activities<br>- Requiring spaces of communication with the managers of the purchase/sales area and spaces to report problems and needs<br>- Encouraging and contributing to the enhancement of local products<br>- Requiring spaces for collaboration and meetings between the stakeholders<br>- Requiring spaces for social aggregation<br>- Requiring training spaces on the enogastronomic and cultural history of the territory	- Participatory structure of the governance of the supply chain, of training and cultural spaces<br>- Spaces of communication with the managers of the purchase/sale area and reporting problems and needs<br>- Spaces aimed at facilitating the construction of collaborative projects between stakeholders<br>- Strategies to enhance local products<br>- Spaces for social gathering<br>- Training spaces about enogastronomical and cultural history of the territory	- Definition of governance based on participatory democracy and co planning of educational and cultural activities<br>- Presence of clear methods of communication with the managers of the purchase/sale area<br>- Presence of clear methods of reporting problems and needs<br>- Aggregation of the offer of local producers<br>- Facilitate strategies for collaboration between the stakeholders<br>- Presence of refreshment points, pubs, a cultural centre and a park<br>- Training courses on the food and wine history of the area

**Freedom**	- Autonomy<br>- Choice<br>- Self esteem<br>- Self affirmation	- Clarity and fairness in contracts<br>- Participatory governance of the supply chain, of training and cultural spaces<br>- Possibility to communicate with the managers of the purchase/sale area and to report problems and needs<br>- Possibility to develop and propose your own ideas around the design of spaces and governance of the supply chain	- Requiring contracts that are clear and respect producers' needs<br>- Requiring spaces for participatory democracy and co planning of educational and cultural activities<br>- Requiring spaces of communication with the managers of the purchase/sales area and spaces to report problems and needs<br>- Requiring spaces to share ideas on the design of spaces and governance of the supply chain at the meeting	- Clear contracts that respect producers' needs<br>- Participatory structure of the governance of the supply chain, of training and cultural spaces<br>- Spaces of communication with the managers of the purchase/sale area and reporting problems and needs<br>- Spaces to share ideas on the design of spaces and governance of the supply chain at the meeting	- Clear contracts that respect producers' needs<br>- Definition of governance based on participatory democracy and the co planning of educational and cultural activities<br>- Presence of clear methods of communication with the managers of the purchase/sale area<br>- Presence of clear methods of reporting problems and needs
**Spirituality**	- Motivation<br>- Consistency with one's personal values<br>- Empathy towards others and nature<br>- Awareness and self knowledge	- Ethical and ecological governance of the supply chain, and according to universal human values<br>- Collaboration and meetings between the stakeholders<br>- Social aggregation<br>- Participatory governance of the supply chain, of training and cultural spaces<br>- Possibility to develop communication skills and empathy<br>- Possibility to acquire the awareness of own limits and potential<br>- Possibility to be present to oneself.	- Requiring ethical and ecological governance of the supply chain and verifying methods consistent with universal human values<br>- Requiring spaces for collaboration and meetings between the stakeholders, social aggregation, participatory democracy and co planning of educational and cultural activities<br>- Requiring training courses on communication skills and empathy, the practices of self knowledge.	- Governance of the supply chain built according to ethical and ecological principles and human values<br>- Participatory certification of the governance<br>- Spaces for collaboration and meetings between the stakeholders,<br>- social aggregation, participatory democracy and co planning of educational and cultural activities<br>- Training courses on communication skills and empathy<br>- Training courses on the practices of self knowledge and spaces used for this purpose.	- Definition of governance built according to ethical and ecological principles and human values<br>- and to human values<br>- Participatory certification of the governance (Common Good Balance Sheet, CSR, etc.)<br>- Strategies to facilitate collaboration between stakeholders<br>- Presence of refreshment points, pubs, a cultural centre and a park<br>- Definition of governance according to participatory democracy and the co planning of educational and cultural activities<br>- Training courses on communication skills and empathy, self awareness practices.

Table S3. Matrix of holders

Holders' needs	Being	Having	Doing	Interacting	Design elements
**Subsistence**	- Physical and mental health<br>- Security	- Fair price<br>- Food quality and wholesomeness<br>- Non-food product quality<br>- Reliability of suppliers and continuity of product deliveries	- Demand transparency on price formation<br>- Obtain guarantees on product quality and continuity of deliveries	- Participatory definition of a guarantee protocol on transparency of suppliers' pricing, the quality of products and continuity of deliveries by suppliers	- A guarantee protocol on transparency of suppliers' pricing, the quality of products and continuity of deliveries by suppliers
**Protection**	- Physical and mental health<br>- Security<br>- Solidarity	- Fair price<br>- Food quality and wholesomeness<br>- Non-food product quality<br>- Reliability of suppliers and continuity of product deliveries<br>- Stakeholder collaboration programmes	- Demand transparency on price formation<br>- Obtain guarantees on product quality and continuity of deliveries<br>- Creating spaces aimed at building collaborative projects: between owners, between owners and producers, between owners and customers and between owners and employees	- Participatory definition of a guarantee protocol on transparency of suppliers' pricing, the quality of products and continuity of deliveries by suppliers<br>- Spaces aimed at building collaborative projects: between owners, between owners and producers, between owners and customers and between owners and employees	- A guarantee protocol on transparency of suppliers' pricing, the quality of products and continuity of deliveries by suppliers<br>- Spaces aimed at building collaborative projects: between owners, between owners and producers, between owners and customers and between owners and employees (co-planning production with producers and customers; participatory price formation; coordination between producers in case of production failures; flexible definition of working hours, etc.).
**Affection**	- Respect<br>- Relationality<br>- Solidarity	- Possibility to report problems and needs to customers, suppliers and employees<br>- Social aggregation, training and culture fruition<br>- Participatory governance of the supply chain<br>- Collaboration and meetings between the stakeholders	- Requiring spaces of communication with the other stakeholders and spaces to report problems and needs<br>- Requiring spaces for social aggregation, training and culture fruition<br>- Requiring the opportunity to participate in the governance of the supply chain<br>- Promoting and activate collaborative projects between stakeholders	- Spaces of communication with the other stakeholders and reporting problems and needs<br>- Spaces for social aggregation, training and cultural frruition<br>- Participatory structure of the governance of the supply chain<br>- Spaces aimed at facilitating the construction of collaborative projects between stakeholders	- Clear methods of communication with the other stakeholders<br>- Spaces for social aggregation, training and cultural frruition<br>- Participatory structure of the governance of the supply chain<br>- Clear methods of reporting problems and needs<br>- Spaces aimed at the construction of collaborative projects between stakeholders

			- Promoting the creation of meeting spaces between stakeholders		
**Understanding**	- Critical consciousness<br>- Knowledge<br>- Curiosity	- Transparency of the production system<br>- Contractual clarity<br>- Lifelong learning	- Require transparency of the production system<br>- Require clarity of contractual relationships<br>- Requiring and promoting spaces for ongoing formation	- Communication transparency protocol of the chain governance<br>- Protocol of understanding on production and delivery methods<br>- Training spaces on production techniques and on healthy and eco-compatible transformation processes<br>- Spaces of permanent training	- Communication transparency protocol of the chain governance<br>- Protocol of understanding on production and delivery methods (certifications, production failures, etc.)<br>- Training spaces on production techniques and on healthy and eco-compatible transformation processes<br>- Definition of a permanent participatory training model
**Participation**	- Relationality<br>- Defense of one's rights and expression of one's personality<br>- Equity<br>- Sense of community	- Social aggregation, training and culture<br>- Participatory governance of the supply chain<br>- Collaboration and meetings between the stakeholders<br>- Possibility to communicate with the other stakeholders and to report problems and needs<br>- Fair price of products<br>- Fair income<br>- Recognition of owners' needs and workers' rights<br>- Possibility of positively influencing the territorial ecosystem and social and relational system<br>- Possibility of positively influencing and supporting the local economy<br>- Possibility of positively influencing awareness and culture in one's own territory	- Requiring spaces for social aggregation, training and culture fruition<br>- Work to a participated governance of the supply chain<br>- Promote the creation of spaces aimed at building collaborative projects between the stakeholders<br>- Promoting and activate collaborative projects between stakeholders<br>- Requiring spaces of communication with the other stakeholders and spaces to report problems and needs<br>- Training on communication skills<br>- Training on participatory democracy models and good assembly practices<br>- Obtain assurances on the fairness of the price<br>- Encouraging a participatory governance model through which to influence the social, economic and environmental processes associated with food<br>- Requiring and promoting spaces for ongoing formation	- Spaces for social gathering, training and cultural dissemination<br>- Participatory structure of the governance of the supply chain<br>- Spaces aimed at facilitating the construction of collaborative projects between stakeholders<br>- Spaces of communication with the other stakeholders and of reporting problems and needs<br>- Participatory definition of a guarantee protocol on the fairness of the price by suppliers<br>- Spaces for training on: communication skills, participatory democracy and assembly techniques	- Presence of refreshment points, pubs, a cultural centre and a park<br>- Definition of a participatory governance model of the supply chain<br>- Presence of spaces used for co designing processes<br>- Strategies to promote meetings and collaborative projects between stakeholders<br>- Presence of clear methods of communication with the other stakeholders<br>- Presence of clear methods of reporting problems and needs<br>- Training courses on: participatory democracy assembly techniques and communication skills<br>- Participatory definition between stakeholders of a guarantee protocol on the fairness of the prices (Contracts protocol and Pact for food, co-production planning with producers and customers; participatory price formation)

					- Multidimensional structure of participatory governance of the supply chain.
**Idleness**	- Relationality<br>- Carefreeness<br>- Fun<br>- Pleasure	- Social aggregation<br>- Lifelong learning<br>- Entertainment (Art and Culture)	- Creating spaces for social gathering<br>- Creating and promoting spaces for ongoing formation<br>- Creating spaces for entertainment	- Spaces for social gathering, training and cultural dissemination<br>- Spaces for entertainment	- Presence of refreshment points, pubs, a cultural centre and a park<br>- Permanent training spaces (training courses, farm visits aiming to know the producers, training trips)<br>- Spaces used for entertainment
**Creation**	- Professional skills<br>- Sensitivity	- Possibility of positively influencing the territorial ecosystem and social and relational system<br>- Possibility of positively influencing and supporting the local economy<br>- Possibility of positively influencing awareness and culture in one’s own territory	- Encouraging a participatory governance model through which to influence the social, economic and environmental processes associated with food<br>- Promoting sustainable local production<br>- Requiring and promoting spaces for ongoing formation	- Environmental regeneration projects<br>- Social regeneration projects<br>- Projects to promote sustainable local production<br>- Training spaces open to the territory	- Environmental regeneration projects<br>- Social regeneration projects<br>- Projects to promote sustainable local production<br>- Training spaces open to the territory
**Identity**	- Self esteem<br>- Self affirmation<br>- Consistency with one’s values<br>- Sense of belonging to the community and the territory	- Recognition of owners' needs<br>- Collaboration and meetings between the stakeholders<br>- Social aggregation<br>- Possibility to promote and enhance foods of the enogastronomic and cultural history of the territory	- Creating spaces for participatory democracy and co planning of educational and cultural activities<br>- Creating spaces of communication with the other stakeholders and spaces to report problems and needs<br>- Encouraging and contributing to the enhancement of local products<br>- Creating spaces for collaboration and meetings between the stakeholders<br>- Creating spaces for social aggregation<br>- Creating training spaces on the enogastronomic and cultural history of the territory	- Participatory structure of the governance of the supply chain, of training and cultural spaces<br>- Spaces of communication with the other actors of the supply chain and reporting problems and needs<br>- Spaces aimed at facilitating the construction of collaborative projects between stakeholders<br>- Strategies to enhance local products<br>- Spaces for social gathering<br>- Training spaces about enogastronomical and cultural history of the territory	- Definition of governance based on participatory democracy and co planning of educational and cultural activities<br>- Presence of clear methods of communication with the other stakeholders<br>- Presence of clear methods of reporting problems and needs<br>- Strategies to facilitate collaboration between supply chain actors (co-design of CSA-model productions; compensation of production failures, use of Umbrex-type trade credit circuits; sharing of personnel and machines between companies, sharing of company waste/circular economy)

					- Facilitate strategies for collaboration between the stakeholders<br>- Presence of refreshment points, pubs, a cultural centre and a park<br>- Training courses on the food and wine history of the area
**Freedom**	- Autonomy<br>- Choice<br>- Self esteem<br>- Self affirmation	- Clarity and fairness in contracts<br>- Possibility to report problems and needs	- Implementing clear contracts that respect the needs of owners<br>- Creating communication spaces with customers, suppliers and employees<br>- Training on tools for communicating one's needs	- Clear contracts respecting owners' needs<br>- Communication spaces with customers, suppliers and employees<br>- Training courses on tools for communicating one's needs	- Clear contracts respecting owners' needs<br>- Communication spaces with customers, suppliers and employees<br>- Training courses on tools for communicating one's needs
**Spirituality**	- Motivation<br>- Consistency with one's personal values<br>- Empathy towards others and nature<br>- Awareness and self knowledge	- Ethical and ecological governance of the supply chain, and according to universal human values<br>- Collaboration and meetings between the stakeholders<br>- Social aggregation<br>- Participatory governance of the supply chain, of training and cultural spaces<br>- Possibility to develop communication skills and empathy<br>- Possibility to acquire the awareness of own limits and potential<br>- Possibility to be present to oneself.	- Requiring ethical and ecological governance of the supply chain and verifying methods consistent with universal human values<br>- Requiring spaces for collaboration and meetings between the stakeholders, social aggregation, participatory democracy and co planning of educational and cultural activities<br>- Requiring training courses on communication skills and empathy, the practices of self knowledge.	- Governance of the supply chain built according to ethical and ecological principles and human values<br>- Participatory certification of the governance<br>- Spaces for collaboration and meetings between the stakeholders, social aggregation, participatory democracy and co planning of educational and cultural activities<br>- Training courses on communication skills and empathy<br>- Training courses on the practices of self knowledge and spaces used for this purpose.	- Definition of governance built according to ethical and ecological principles and human values and to human values<br>- Participatory certification of the governance (Common Good Balance Sheet, CSR, etc.)<br>- Strategies to facilitate collaboration between stakeholders<br>- Presence of refreshment points, pubs, a cultural centre and a park<br>- Definition of governance according to participatory democracy and the co planning of educational and cultural activities<br>- Training courses on communication skills and empathy, self awareness practices.

Table S4. Matrix of employees

Employees' needs	Being	Having	Doing	Interacting	Design elements
**Subsistence**	- Physical and mental health<br>- Security<br>- Pleasantness of the workplace	- Contracts stable over time<br>- Fair income<br>- Fair work duties<br>- Respect for workers' rights (safety at work, holidays, maternity, etc.)<br>- Healthiness and architectural accessibility of work spaces<br>- Logistical accessibility and proximity to one's place of work<br>- Sustainability of working hours<br>- Flexible working hours<br>- Contact with natural elements	- Obtain stability of contracts<br>- Obtain fairness of remuneration paid and tasks required<br>- Obtain respect for workers' rights<br>- To be trained in bio-architecture and architectural accessibility<br>- Demand optimal bio-architectural design of work spaces<br>- Demand the elimination of architectural barriers<br>- Obtain opening of the workplace at a maximum distance of 15 km<br>- Obtain sustainable and flexible working hours<br>- Train on green space design<br>- Demand optimal green space design of outdoor spaces	- Participatory definition of an owner/employee guarantee protocol on employee rights, continuity of contracts, fairness of the tasks required and of the remuneration paid<br>- Training spaces on bio-architecture and accessibility<br>- Participatory design of work spaces (healthiness and accessibility of environments)<br>- Working place at a maximum distance of 15 km<br>- Sustainable and flexible working hours<br>- Training spaces on green design<br>- Participatory green design of outdoor spaces	- Participatory definition of an owner/employee guarantee protocol on employee rights, continuity of contracts, fairness of the tasks required and of the remuneration paid<br>- Training spaces on bio-architecture and accessibility<br>- Participatory design of work spaces (healthiness and accessibility of environments)<br>- *Food Market* supermarket model (logistical and temporal accessibility, variability of products that can be purchased)<br>- Training courses on green design available to employees<br>- Strategies for involving employees in the co-design of the external spaces of the point of sale and purchase
**Protection**	- Physical and mental health<br>- Security<br>- Solidarity	- Contracts stable over time<br>- Fair income<br>- Fair work duties<br>- Respect for workers' rights (safety at work, holidays, maternity, etc.)<br>- Healthiness and architectural accessibility of work spaces<br>- Stakeholder collaboration programmes	- Obtain stability of contracts<br>- Obtain fairness of remuneration paid and tasks<br>- Obtain respect for workers' rights<br>- To be trained in bio-architecture and architectural accessibility<br>- Demand optimal bio-architectural design of work spaces<br>- Demand the elimination of architectural barriers<br>- Obtain guarantees on the presence of spaces aimed at building collaborative projects: between employees, between employees and owners, between employees and	- Participatory definition of an owner/employee guarantee protocol on employees' rights, continuity of contracts, fairness of the tasks required and of the remuneration paid<br>- Training spaces on bio-architecture and accessibility<br>- Participatory design of work spaces (healthiness and accessibility of environments)<br>- Spaces aimed at building collaborative projects: between employees, between employees and owners, between	- Participatory definition of an owner/employee guarantee protocol on employees' rights, continuity of contracts, fairness of the tasks required and of the remuneration paid<br>- Training spaces on bio-architecture and accessibility<br>- Participatory design of work spaces (healthiness and accessibility of environments)<br>- Spaces aimed at building collaborative projects: between employees, between employees and owners, between employees and producers and between employees and customers

			producers and between employees and customers	employees and producers and between employees and customers	(co-management of sales areas with owners and customers; flexible coverage of working time in collaboration with other employees, etc.).
**Affection**	- Respect<br>- Relationality<br>- Solidarity	- Possibility to report problems and needs to those responsible for the workplace<br>- Social aggregation, training and culture fruition<br>- Participatory governance of the supply chain<br>- Collaboration and meetings between the stakeholders	- Requiring spaces and clear modality of communication with the other stakeholders and spaces to report problems and needs<br>- Requiring spaces for social aggregation, training and culture fruition<br>- Requiring the opportunity to participate in the governance of the supply chain<br>- Promoting and activate collaborative projects between stakeholders<br>- Promoting the creation of meeting spaces between stakeholders	- Spaces of communication with the other stakeholders and reporting problems and needs<br>- Spaces for social aggregation, training and cultural frruition<br>- Participatory structure of the governance of the supply chain<br>- Spaces aimed at facilitating the construction of collaborative projects between stakeholders	- Clear methods of communication with the other stakeholders<br>- Clear methods of reporting problems and needs<br>- Spaces of communication with the other stakeholders and reporting problems and needs<br>- Spaces for social aggregation, training and cultural frruition<br>- Participatory structure of the governance of the supply chain<br>- Spaces aimed at the construction of collaborative projects between stakeholders
**Understanding**	- Critical consciousness<br>- Knowledge<br>- Curiosity	- Transparency of the workplace governance<br>- Contractual clarity<br>- Lifelong learning	- Require transparency of the of workplace governance processes<br>- Require clarity of contractual relationships<br>- Requiring and promoting spaces for ongoing formation	- Communication transparency protocol on supply chain governance<br>- Training spaces on production techniques and on healthy and eco-compatible transformation processes<br>- Spaces of permanent training	- Communication transparency protocol of the chain governance<br>- Training spaces on production techniques and on healthy and eco-compatible transformation processes<br>- Definition of a permanent participatory training model

**Participation**	- Relationality<br>- Defense of one's rights and expression of one's personality<br>- Equity<br>- Sense of community	- Social aggregation, training and culture<br>- Participatory governance of the supply chain<br>- Collaboration and meetings between the stakeholders<br>- Possibility to communicate with the other stakeholders and to report problems and needs<br>- Contracts stable over time<br>- Fair income<br>- Recognition of employees' needs and workers' rights<br>- Possibility of positively influencing the territorial ecosystem and social and relational system<br>- Possibility of positively influencing and supporting the local economy<br>- Possibility of positively influencing awareness and culture in one's own territory	- Requiring spaces for social aggregation, training and culture fruition<br>- Requiring the possibility to participate to the *governance* of the supply chain<br>- Promote the creation of spaces aimed at building collaborative projects between the stakeholders<br>- Promoting and activate collaborative projects between stakeholders<br>- Requiring spaces of communication with the other stakeholders and spaces to report problems and needs<br>- Training on communication skills<br>- Training on participatory democracy models and good assembly practices<br>- Obtain assurances on the stability of contracts<br>- Obtaining guarantees on compensation paid and fairness of work tasks<br>- Encouraging a participatory governance model through which to influence the social, economic and environmental processes associated with food<br>- Requiring and promoting spaces for ongoing formation	- Spaces for social gathering, training and cultural dissemination<br>- Participatory structure of the governance of the supply chain<br>- Spaces aimed at facilitating the construction of collaborative projects between stakeholders<br>- Spaces of communication with the other stakeholders and of reporting problems and needs<br>- Participatory definition of a guarantee protocol on the stability of contracts<br>- Spaces for training on: communication skills, participatory democracy and assembly techniques	- Presence of refreshment points, pubs, a cultural centre and a park<br>- Definition of a participatory governance model of the supply chain<br>- Presence of spaces used for co designing processes<br>- Strategies to promote meetings and collaborative projects between stakeholders<br>- Presence of clear methods of communication with the other stakeholders<br>- Presence of clear methods of reporting problems and needs<br>- Training courses on: participatory democracy assembly techniques and communication skills<br>- Participatory definition between employees and owners of a guarantee protocol on stability of contracts, fairness of work tasks and remuneration paid<br>- Multidimensional structure of participatory governance of the supply chain.

**Idleness**	- Relationality<br>- Carefreeness<br>- Fun<br>- Pleasure	- Social aggregation<br>- Lifelong learning<br>- Entertainment (Art and Culture)	- Creating spaces for social gathering<br>- Creating and promoting spaces for ongoing formation<br>- Creating spaces for entertainment	- Spaces for social gathering, training and cultural dissemination<br>- Spaces for entertainment	- Presence of refreshment points, pubs, a cultural centre and a park<br>- Permanent training spaces (training courses, farm visits aiming to know the producers, training trips)<br>- Spaces used for entertainment
**Creation**	- Beauty<br>- Harmony<br>- Fantasy<br>- Collective well-being	- Contact with nature<br>- Aesthetically pleasing work and outdoor spaces<br>- Opportunities for cultural growth (Art and Culture)<br>- Freedom to develop and propose one's own ideas around the design of spaces and the governance of the chain<br>- Participatory governance of the supply chain, training and cultural spaces	- Demand optimal landscaping of outdoor spaces<br>- Demand harmonious and aesthetically pleasing architectural design of workspaces<br>- Demand spaces for cultural events<br>- Demand the freedom to develop and present their own ideas on the design of spaces and the governance of the supply chain at the meeting<br>- Demand spaces for participatory democracy and co-design of educational and cultural activities	- Participatory design of green spaces outside the work space<br>- Harmonious and beautiful architectural design of the shopping/sales areas<br>- Spaces for cultural events<br>- Spaces of participatory democracy and co-planning of educational and cultural activities	- Strategies for involving customers in the co-design of the interior and exterior spaces of the point of sale and purchase<br>- Architecturally pleasing design of work spaces (in terms of beauty, functionality, relationality, harmony etc.)<br>- Spaces for cultural events (concerts, film shows, theatre performances, exhibitions, book presentations etc.)<br>- Definition of governance based on participatory democracy and co-design of training and cultural activities<br>- Spaces for participatory democracy sessions
**Identity**	- Self esteem<br>- Self affirmation<br>- Consistency with one’s values<br>- Sense of belonging to the community and the territory	- Participatory governance of the supply chain, training and cultural spaces<br>- Communicative accessibility to workplace managers<br>- Ability to report problems and needs<br>- Collaboration and meetings between the stakeholders<br>- Social aggregation	- Demand spaces for participatory democracy in the chain and co-design of training and cultural activities<br>- Obtain assurances on how to communicate with those in charge of the workspace<br>- Request spaces for reporting problems and needs<br>- Demand spaces for collaboration between actors in the sector<br>- Demand spaces for social aggregation	- Spaces of participatory democracy of the chain and co-design of training and cultural activities<br>- Presence of clear ways of communicating with the workplace managers<br>- Presence of spaces for reporting problems and needs<br>- Spaces aimed at facilitating the construction of collaborative projects between stakeholders<br>- Strategies to enhance local products	- Definition of governance based on participatory democracy and co planning of educational and cultural activities<br>- Presence of clear methods of communication with the other stakeholders<br>- Presence of clear methods of reporting problems and needs<br>- Strategies to facilitate collaboration between supply chain actors (co-management of workspaces with owners and clients; flexible coverage

				- Spaces for social gathering	of working time in collaboration with other employees, etc.)<br>- Presence of refreshment points, pubs, a cultural centre and a park
**Freedom**	- Autonomy<br>- Choice<br>- Self esteem<br>- Self affirmation	- Clarity and fairness in contracts<br>- Participatory governance of the supply chain, training spaces and cultural spaces<br>- Freedom of communication with workplace managers<br>- Possibility to report problems and needs	- Demand contracts that are clear and respect employees' needs<br>- Demand spaces for participatory democracy and co-design of training and cultural activities<br>- Obtaining guarantees on how to communicate with those responsible for the workplace<br>- Demand spaces for reporting problems and needs	- Clear contracts respecting employees' needs<br>- Spaces for participatory democracy and co-planning of training and cultural activities<br>- Presence of clear communication modalities with the workplace managers<br>- Spaces for reporting problems and needs	- Clear contracts respecting employees' needs<br>- Spaces for participatory democracy and co-planning of training and cultural activities<br>- Presence of clear communication modalities with the workplace managers<br>- Spaces for reporting problems and needs
**Spirituality**	- Motivation<br>- Consistency with one's personal values<br>- Empathy towards others and nature<br>- Awareness and self knowledge	- Ethical and ecological governance of the supply chain, and according to universal human values<br>- Collaboration and meetings between the stakeholders<br>- Social aggregation<br>- Participatory governance of the supply chain, of training and cultural spaces<br>- Possibility to develop communication skills and empathy<br>- Possibility to acquire the awareness of own limits and potential<br>- Possibility to be present to oneself.	- Requiring ethical and ecological governance of the supply chain and verifying methods consistent with universal human values<br>- Requiring spaces for collaboration and meetings between the stakeholders, social aggregation, participatory democracy and co planning of educational and cultural activities<br>- Requiring training courses on communication skills and empathy, the practices of self knowledge.	- Governance of the supply chain built according to ethical and ecological principles and human values<br>- Participatory certification of the governance<br>- Spaces for collaboration and meetings between the stakeholders, social aggregation, participatory democracy and co planning of educational and cultural activities<br>- Training courses on communication skills and empathy<br>- Training courses on the practices of self knowledge and spaces used for this purpose.	- Definition of governance built according to ethical and ecological principles and human values<br>- and to human values<br>- Participatory certification of the governance (Common Good Balance Sheet, CSR, etc.)<br>- Strategies to facilitate collaboration between stakeholders<br>- Presence of refreshment points, pubs, a cultural centre and a park<br>- Definition of governance according to participatory democracy and the co planning of educational and cultural activities<br>- Training courses on communication skills and empathy, self awareness practices.

Table S5. Matrix of collectivity

Collectivity needs	Being	Having	Doing	Interacting	Design elements
**Subsistence**	- Physical and mental health<br>- Security	- Satisfaction of food needs<br>- Eco-sustainable production systems (i.e. promoters of ecosystem services, control of the carbon footprint, use of renewable energy sources)<br>- Healthiness and accessibility of spaces for the agri-food sector<br>- Virtuous economic context (aimed at increasing employment, promoting youth employment, fair remuneration, protection of workers' rights)<br>- Social aggregation and relationality	- Implement food resilience policies<br>- Promote and support environmentally friendly production processes<br>- Promote healthy standards in agro-food spaces<br>- Promote the accessibility of spaces in the agro-food sector<br>- Promote virtuous economic processes<br>- Promote social aggregation and active participation in local initiatives through entertainment programmes	- Co-designing policies for territorial food resilience<br>- Information and training programmes on the sustainability of the food chain, healthy eating, critical consumption, ecology<br>- Training programmes on spatial planning and green design<br>- Urban planning that guarantees physical accessibility to points of sale<br>- Policies to support employment, worker protection and fair pay for work<br>- Control systems for contractual fairness<br>- Promotion of co-designed circuits for periodic programming of entertainment and cultural events	- Co-designing policies for territorial food resilience<br>- Information and training programmes on the sustainability of the food chain, healthy eating, critical consumption, ecology<br>- Training programmes on spatial planning and green design<br>- Urban planning that guarantees physical accessibility to points of sale<br>- Policies to support employment, worker protection and fair pay for work<br>- Control systems for contractual fairness<br>- Co-designed circuits for periodic programming of entertainment and cultural events
**Protection**	- Care and health<br>- Care and regeneration of the environment<br>- Solidarity	- Meeting food needs<br>- Healthiness and accessibility of spaces for the agri-food sector<br>- Social aggregation and relationality<br>- Healthiness of agri-food premises<br>- Virtuous production systems (i.e. promoting ecosystem services, controlling the carbon	- Implementing food resilience policies<br>- Promoting critical consumption and combating food waste<br>- Promote training programmes on critical consumption, sustainable agriculture techniques and ecology<br>- Promote and verify standards of healthiness and architectural accessibility of agri-food spaces<br>- Promote social aggregation and active participation in initiatives in	- Territorial food resilience co-design policies<br>- Information and training programmes on the sustainability of the food supply chain, healthy eating, critical consumption, ecology<br>- Urban planning that guarantees the physical accessibility of the points of sale<br>- Promotion of co-planned circuits for the periodic	- Territorial food resilience co-design policies<br>- Information and training programmes on the sustainability of the food supply chain, healthy eating, critical consumption, ecology<br>- Urban planning that guarantees the physical accessibility of the points of sale<br>- Co-planned circuits for the periodic programming of entertainment and cultural events

		footprint, using renewable energy sources)<br>- Minimisation of food waste<br>- Land regeneration programmes<br>- Support policies for the most vulnerable social groups<br>- Enhancement and promotion of cultural biodiversity (customs, traditions, flavours, landscapes, etc.)	the area, through entertainment programmes<br>- Developing systems and protocols for monitoring the environmental conformity of production processes<br>- Promote territorial regeneration projects<br>- Implementing support policies for the most vulnerable social groups<br>- nhancing and promoting cultural biodiversity	programming of entertainment and cultural events<br>- Urban planning physical accessibility to points of sale<br>- Development of systems and protocols for monitoring the environmental conformity of production processes<br>- Awareness campaigns against food waste and critical consumption<br>- Co-design of territorial regeneration interventions<br>- Promotion of co-designed circuits for the regular programming of entertainment and cultural events<br>- Facilitation of access to the supply chain for weaker social groups (e.g. meal vouchers)<br>- Campaigns to promote cultural biodiversity	- Urban planning physical accessibility to points of sale<br>- Systems and protocols for monitoring the environmental conformity of production processes<br>- Awareness campaigns against food waste and critical consumption<br>- Territorial regeneration interventions<br>- Co-designed circuits for the regular programming of entertainment and cultural events<br>- Facilitation of access to the supply chain for weaker social groups (e.g. meal vouchers)<br>- Campaigns to promote cultural biodiversity
**Affection**	- Respect<br>- Relationality<br>- Solidarity	- Communicative accessibility<br>- Protection of workers' rights<br>- Ability to report problems and needs<br>- Social aggregation, training and culture fruition<br>- Enhancement and promotion of cultural biodiversity (customs, traditions, flavours, landscapes, etc.)	- Promoting the development of communication spaces<br>- Guaranteeing the protection of workers' rights<br>- Guaranteeing spaces and ways of reporting problems and needs<br>- Promoting social aggregation and active participation in initiatives in the area, through entertainment programmes<br>- Enhancing and promoting cultural biodiversity<br>- Implementing support policies for the most vulnerable social groups	- Spaces for communication<br>- Development of protection protocols for workers with regard to contractual conditions<br>- Spaces for reporting problems and needs<br>- Promotion of co-planned circuits for the periodic programming of entertainment and cultural events<br>- Spaces for social aggregation, training and cultural frruition<br>- Campaigns to promote cultural biodiversity	- Spaces for communication<br>- Protection protocols for workers with regard to contractual conditions<br>- Spaces for reporting problems and needs<br>- Co-planned circuits for the periodic programming of entertainment and cultural events<br>- Spaces for social aggregation, training and cultural frruition<br>- Campaigns to promote cultural biodiversity<br>- Facilitating access to the supply chain for weaker social groups (e.g. meal vouchers)

		- Support policies for the most vulnerable social groups		- Facilitating access to the supply chain for weaker social groups (e.g. meal vouchers)	
**Understanding**	- Critical consciousness<br>- Knowledge<br>- Curiosity	- Communicative accessibility<br>- Opportunities for training and cultural deepening<br>- Enhancement and promotion of cultural biodiversity (customs, traditions, flavours, landscapes, etc.)	- Promoting the development of communication spaces<br>- Promoting training on critical consumption, the sustainability of the food chain and ecology<br>- Enhancing and promoting cultural biodiversity	- Communication spaces<br>- Spaces dedicated to training and cultural development<br>- Campaigns to promote cultural biodiversity	- Communication spaces<br>- Spaces dedicated to training and cultural development<br>- Campaigns to promote cultural biodiversity
**Participation**	- Relationality<br>- Defense of one's rights and expression of one's personality<br>- Equity	- Social aggregation, training and culture<br>- Communicative accessibility<br>- Enhancement and promotion of cultural biodiversity (customs, traditions, flavours, landscapes, etc.)<br>- Ability to report problems and needs<br>- Participatory spatial planning programmes and co-design of supply systems<br>- Virtuous economic environment (aimed at increasing employment, promoting youth employment, fair compensation, protection of workers' rights)	- Promoting social aggregation and active participation in initiatives in the area, through entertainment programmes<br>- Promoting the development of communication spaces<br>- Enhancing and promoting cultural biodiversity<br>- Providing spaces and ways of reporting problems and needs<br>- Ensure training on models of participatory democracy and good assembly practices<br>- Promoting virtuous economic processes	- Promotion of co-planned circuits for the periodic programming of entertainment and cultural events<br>- Spaces dedicated to social aggregation, relationality and cultural development<br>- Communication spaces<br>- Campaigns to promote cultural biodiversity<br>- Training spaces on tools for communicating needs<br>- Spaces for reporting problems and needs<br>- Training spaces on participatory democracy and assembly techniques<br>- Policies in support of employment, worker protection and fair pay for work<br>- Development of protection protocols for workers with regard to contractual conditions	- Co-planned circuits for the periodic programming of entertainment and cultural events<br>- Spaces dedicated to social aggregation, relationality and cultural development<br>- Communication spaces<br>- Campaigns to promote cultural biodiversity<br>- Training spaces on tools for communicating needs<br>- Spaces for reporting problems and needs<br>- Training spaces on participatory democracy and assembly techniques<br>- Policies in support of employment, worker protection and fair pay for work<br>- Development of protection protocols for workers with regard to contractual conditions

**Idleness**	- Relationality<br>- Carefreeness<br>- Fun<br>- Pleasure	- Social aggregation<br>- Entertainment (Art and Culture)	- Promoting social aggregation and active participation in local initiatives through entertainment programmes<br>- Encouraging the realisation of opportunities for social aggregation, training and cultural deepening	- Promotion of co-designed circuits for the periodic programming of entertainment and cultural events<br>- Spaces dedicated to social aggregation, training and cultural development	- Co-designed circuits for the periodic programming of entertainment and cultural events<br>- Spaces dedicated to social aggregation, relationality and cultural learning
**Creation**	- Professional skills<br>- Sensitivity<br>- Beauty<br>- Harmony<br>- Fantasy	- Interventions to protect or restore the territorial ecosystem<br>- Interventions to improve the territorial, social and relational system, and territorial economy<br>- Interventions to raise awareness of awareness and culture in one's own territory<br>- Opportunities for cultural growth (Art and Culture)<br>- Freedom to contribute to the design of spaces and the governance of the territory	- Promoting environmental regeneration projects<br>- Promoting social aggregation projects<br>- Promoting sustainable local production<br>- Promoting training spaces open to the territory<br>- Encouraging the creation of opportunities for social aggregation, training and cultural development<br>- Supporting participatory planning in the local area	- Projects of environmental regeneration, social aggregation and of valorization of local sustainable productions<br>- Training spaces open to the territory<br>- Spaces dedicated to social gathering, training and cultural development<br>- Training spaces on participatory democracy and assembly techniques<br>- Spaces devoted to participatory governance of the territory	- Projects of environmental regeneration, social aggregation and of valorization of local sustainable productions<br>- Training spaces open to the territory<br>- Spaces dedicated to social gathering, training and cultural development<br>- Training spaces on participatory democracy and assembly techniques<br>- Spaces devoted to participatory governance of the territory
**Identity**	- Self esteem<br>- Self affirmation<br>- Consistency with one's values<br>- Sense of belonging to the community and the territory	- Communicative accessibility<br>- Ability to report problems and needs<br>- Participatory spatial planning programmes and co-design of supply systems<br>- Interventions to protect or restore the territorial ecosystem<br>- Interventions to improve the territorial social and relational system	- Promoting the development of communication spaces<br>- Guaranteeing spaces and methods for reporting problems and needs<br>- Ensure training on models of participatory democracy and good assembly practices<br>- Promoting environmental regeneration projects<br>- Promoting social aggregation projects<br>- Promoting sustainable local production	- Communication spaces<br>- Spaces for training on tools for communicating needs<br>- Spaces for reporting problems and needs<br>- Spaces for training on participatory democracy and assembly techniques<br>- Environmental regeneration projects<br>- Social aggregation projects<br>- Projects for the promotion of sustainable local products	- Communication spaces<br>- Spaces for training on tools for communicating needs<br>- Spaces for reporting problems and needs<br>- Spaces for training on participatory democracy and assembly techniques<br>- Environmental regeneration projects<br>- Social aggregation projects<br>- Projects for the promotion of sustainable local products<br>- Training spaces open to the territory

		- Interventions to improve the territorial economy<br>- Interventions to raise awareness of awareness and culture in one's own territory	- Promoting training spaces open to the territory	- Training spaces open to the territory	
**Freedom**	- Autonomy<br>- Choice<br>- Self esteem<br>- Self affirmation	- Communicative accessibility<br>- Ability to report problems and needs<br>- Participatory spatial planning programmes and co-design of supply systems<br>- Freedom to contribute to spatial design and land governance	- Promoting the development of communication spaces<br>- Guaranteeing spaces and ways of reporting problems and needs<br>- Guarantee training on models of participatory democracy and good assembly practices<br>- Support participatory planning in the area	- Communication spaces<br>- Spaces for training on tools for communicating needs<br>- Spaces for reporting problems and needs<br>- Training spaces on participatory democracy and assembly techniques<br>- Spaces for participatory governance of the territory	- Communication spaces<br>- Spaces for training on tools for communicating needs<br>- Spaces for reporting problems and needs<br>- Training spaces on participatory democracy and assembly techniques<br>- Spaces for participatory governance of the territory
**Spirituality**	- Motivation<br>- Consistency with one's personal values<br>- Empathy towards others and nature<br>- Awareness and self knowledge	- Ethical and ecological governance of the supply chain, and according to universal human values<br>- Social aggregation and relationality<br>- Enhancement and promotion of cultural biodiversity (customs, traditions, flavours, landscapes, etc.)<br>- Interventions for the protection or restoration of the territorial ecosystem<br>- Interventions to improve the social and relational territorial system<br>- Awareness of the limits and potentialities of the human being<br>- Presence to oneself.	- Promoting training in ethical issues and universal human values<br>- Encourage policies and modes of governance, as well as its review, consistent with universal human values<br>- Promoting social aggregation and active participation in initiatives in the area, through entertainment programmes<br>- Enhancing and promoting cultural biodiversity<br>- Promoting environmental regeneration projects<br>- Promoting social aggregation projects<br>- Encouraging training courses on self-knowledge practices	- Spaces for training on ethical issues and universal human values<br>- Participatory certification of governance according to universal human values<br>- Promotion of co-designed circuits for regular programming of entertainment and cultural events<br>- Campaigns to promote cultural biodiversity<br>- Environmental and social regeneration projects<br>- Self-awareness campaigns<br>- Training courses on self-awareness practices	- Spaces for training on ethical issues and universal human values<br>- Participatory certification of governance according to universal human values (application of Common Good Budgeting, CSR, etc.)<br>- Co-designed circuits for regular programming of entertainment and cultural events<br>- Campaigns to promote cultural biodiversity<br>- Environmental and social regeneration projects<br>- Self-awareness campaigns<br>- Training courses on self-awareness practices

Table S6. The clustered design elements

	Consumers	Producer	Holder	Employee	Collectivity	Overall design elements
**Values/ principles**	– Ethical and ecological governance of the supply chain, and according to universal human values.<br>– Participatory certification of the governance (Common Good Balance Sheet, CSR, etc.).	– Ethical and ecological governance of the supply chain, and according to universal human values<br>– Participatory certification of the governance (Common Good Balance Sheet, CSR, etc.).	– Ethical and ecological governance of the supply chain, and according to universal human values<br>– Participatory certification of the governance (Common Good Balance Sheet, CSR, etc.).	– Ethical and ecological governance of the supply chain, and according to universal human values<br>– Participatory certification of the governance (Common Good Balance Sheet, CSR, etc.).	– Participatory certification of the governance (Common Good Balance Sheet, CSR, etc.).	– Ethical and ecological governance of the supply chain, and according to universal human values<br>– Participatory certification of the governance (Common Good Balance Sheet, CSR, etc.).
**Governance/ training model**	– Definition of governance according to participatory democracy and the co planning of educational and cultural activities<br>– Definition of a model of ongoing participatory training<br>– Multidimensional structure of participatory governance of the supply chain	– Definition of governance according to participatory democracy and the co planning of educational and cultural activities<br>– Definition of a model of ongoing participatory training<br>– Multidimensional structure of participatory governance of the supply chain	– Definition of governance according to participatory democracy and the co planning of educational and cultural activities<br>– Definition of a model of ongoing participatory training<br>– Multidimensional structure of participatory governance of the supply chain	– Definition of governance according to participatory democracy and the co planning of educational and cultural activities<br>– Definition of a model of ongoing participatory training<br>– Multidimensional structure of participatory governance of the supply chain	– Definition of a model of participatory management of public affairs<br>– Definition of a model of ongoing participatory training	– Definition of governance according to participatory democracy and the co planning of educational and cultural activities<br>– Definition of a model of ongoing participatory training<br>– Multidimensional structure of participatory governance of the supply chain
**Training**	– Training courses on:<br>1. human nutrition<br>2. healthy and environmentally friendly production techniques, transformation	– Training courses on:<br>1. healthy and environmentally friendly production techniques, transformation processes and methods of marketing<br>2. green design	– Training courses on:<br>1. healthy and environmentally friendly production techniques, transformation processes and methods of marketing	– Training courses on:<br>1. bio-architecture and architectural accessibility<br>2. green design<br>3. ecology and ecological indicators	– Training courses on:<br>1. sustainability of the food chain, healthy nutrition, critical consumption and ecology	– Training courses on:<br>1. human nutrition<br>2. healthy and environmentally friendly production techniques, transformation processes, and marketing methods

	processes and marketing methods<br>3. bio-architecture and architectural accessibility<br>4. green design<br>5. ecology and ecological indicators<br>6. food and wine history of the area<br>7. communication tools for one's own needs, participatory democracy and assembly techniques<br>8. communication skills and empathy<br>9. self awareness practices<br>– Permanent training spaces (training courses, farm visits aiming to know the producers, training trips).	3. ecology and ecological indicators<br>4. food and wine history of the area<br>5. communication tools for one's own needs, participatory democracy and assembly techniques<br>6. communication skills and empathy<br>7. self awareness practices<br>– Permanent training spaces (training courses, farm visits aiming to know the producers, training trips).	2. bio-architecture and architectural accessibility<br>3. green design<br>4. ecology and ecological indicators<br>5. communication tools for one's own needs, participatory democracy and assembly techniques<br>6. communication skills and empathy<br>7. self awareness practices<br>– Permanent training spaces (training courses, farm visits aiming to know the producers, training trips).	4. communication tools for one's own needs, participatory democracy and assembly techniques<br>5. communication skills and empathy<br>6. self awareness practices<br>– Permanent training spaces (training courses, farm visits aiming to know the producers, training trips).	2. bio-architecture and architectural accessibility<br>3. territorial planning and green design<br>4. self awareness practices<br>– Permanent training spaces (training courses, farm visits aiming to know the producers, training trips).	3. bio-architecture and architectural accessibility<br>4. green design<br>5. ecology and ecological indicators<br>6. food and wine history of the area<br>7. communication tools for one's own needs, participatory democracy and assembly techniques<br>8. communication skills and empathy<br>9. self awareness practices<br>– Permanent training spaces (training courses, farm visits aiming to know the producers, training trips).

**Spaces**	– Spaces used for:<br>1. Food Market<br>2. for training<br>3. social aggregation (refreshment points, pubs, cultural centers, park)<br>4. entertainment<br>5. co design<br>6. reporting problems and needs, as well as for proposing one's own ideas<br>– Bio-architecture building standards: natural construction materials, renewable energy sources, etc.<br>– Strategies for involving customers in the co-design of the internal and external spaces of Food Market.	– Spaces used for:<br>1. sale of experimental products<br>2. meeting between producers and consumers<br>3. training<br>4. co-design<br>5. social aggregation (refreshment points, pubs, cultural centers, park)<br>6. entertainment<br>7. report problems and needs, as well as to propose their own ideas<br>– Strategies for involving producers in the co-design of work spaces<br>– Bio-architecture building standards: natural construction materials, renewable energy sources, etc.	– Spaces used for:<br>1. distribution of products and communication of cultural history of the area<br>2. communication with customers, suppliers and employees<br>3. training<br>4. social aggregation (refreshment points, pubs, cultural centers, parks, etc.)<br>5. entertainment<br>6. reporting problems and needs, as well as for proposing one's own ideas<br>– Strategies for involving holders in the co-design of work spaces<br>– Bio-architecture building standards: natural construction materials, renewable energy sources, etc.	– Spaces used for:<br>1. Food Market<br>2. training<br>3. co-design<br>4. reporting problems and needs, as well as for proposing one's own ideas<br>5. social aggregation (refreshment points, pubs, cultural centers, park)<br>6. entertainment<br>– Strategies for involving employees in the co design of work spaces<br>– Bio-architecture building standards: natural construction materials, renewable energy sources, etc.	– Spaces used for:<br>1. training<br>2. communication<br>3. reporting problems and needs<br>4. participatory democracy and assembly techniques<br>5. social aggregation, relationality and cultural deepening<br>6. training and information on ethics and universal human values.	– Spaces used for:<br>1. Food Market<br>2. sale of experimental products<br>3. distribution of products and communication of cultural history of the area<br>4. meeting and communication<br>5. training<br>6. training and information on ethics and universal human values<br>7. social aggregation (refreshment points, pubs, cultural centres, park)<br>8. entertainment<br>9. co-design<br>10. participatory democracy and assembly techniques<br>11. reporting problems and needs<br>– Strategies for involving stakeholders in the co design of the spaces<br>– Bio-architecture building standards: natural construction materials, renewable energy sources, etc.
**Comunication**	– Clear methods of communication with the managers of the purchase/sale area	– Clear methods of communication with the managers of the purchase/sale area	– Participatory communication transparency protocol on the governance of the supply chain.	– Clear methods of communication with the managers of the purchase/sale area	– Opportunities' promotion for discussion, which facilitate the communication community, and	– Clear methods of communication with the site managers<br>– Clear ways of reporting problems and needs

	– Clear methods of reporting problems and needs<br>– Definition of meeting ways between producers and consumers<br>– Communication transparency protocol on the governance of the production chain.	– Clear methods of reporting problems and needs<br>– Communication transparency protocol of the production chain.		– Clear methods of reporting problems and needs<br>– Communication transparency protocol of the production chain.	territory problems and needs' reporting (assembly sessions, listening days, debates)<br>– Implementation of a manual on communication and reporting methods, for greater communication transparency.	– Participatory communication transparency protocol concerning the governance of the supply chain.
**Cooperation**	– Strategies to promote meetings and collaborative projects between stakeholders (co-design of CSA model productions, customer purchasing groups for products not sold at the point of purchase/sale; sharing of personnel and machines between farms, sharing of farm waste according to the circular economy)<br>– Participatory definition strategies of:<br>1. products of the supply chain and participatory certification of local farmers products<br>2. ecological standards of production and marketing, as well as	– Strategies to promote meetings and collaborative projects between stakeholders (co-design of CSA model productions, customer purchasing groups for products not sold at the point of purchase/sale; sharing of personnel and machines between farms, sharing of farm waste according to the circular economy).	– Strategies to promote meetings and collaborative projects between stakeholders (co-design of production with producers and customers; participatory pricing training; coordination between producers for production failures; flexible definition of working hours, etc.).	– Strategies to promote meetings and collaborative projects between stakeholders (co-management of sales spaces with owners and customers; flexible coverage of working hours in collaboration with other employees, etc.).	– Co-design policies of territorial food resilience<br>– Co-designed circuits for the periodic programming of entertainment and cultural events	– Strategies to promote meetings and collaborative projects between stakeholders (co-design of CSA model productions, customer purchasing groups for products not sold at the point of purchase/sale; sharing of personnel and machines between farms, sharing of farm waste according to the circular economy)<br>– Participatory definition strategies:<br>1. products of the supply chain and participatory certification of local farmers products<br>2. ecological standards of production and marketing, as well as the reduction of food waste

	the reduction of food waste<br>3. prices of a basic products basket<br>4. territorial regeneration projects.					3. prices of a basic products basket<br>4. territorial regeneration projects.<br>5. contractual conditions<br>– Co design policies of territorial food resilience.
**Characterizing elements**	– Careful selection of products and suppliers, according to consumer preferences<br>– Home economics goods<br>– Online ordering and home delivery platform<br>– Aggregation of the offer of local producers.	– Participatory definition between producers and holders of a guarantee protocol on the continuity of contracts, on the equity of the requested productions and on the remuneration paid (Protocol of contracts and Pact for food, co planning of production with owners and customers; participatory price formation)<br>– Memorandum of understanding on the production and transfer methods (certifications, production failures, etc.)<br>– Aggregation of the offer of local producers<br>– Promotion of typical products and local food traditions<br>– Clear contracts that respect the needs of producers.	– Guarantee protocol on the transparency of price formation by suppliers<br>– Protocol on the quality of products and on the continuity of deliveries by suppliers<br>– Clear contracts that respect the owners' needs<br>– Environmental and social regeneration projects<br>– Projects for enhancing sustainable local production.	– Definition of participatory holders/employees of a guarantee protocol on employee rights, on the continuity of contracts, on the equity of the duties required and the remuneration paid<br>– Clear contracts that respect the needs of employees<br>– Clarity of contractual relationships.	– Policies to support employment, worker protection and fair pay for work<br>– Urban planning guaranteeing physical accessibility to the points of sale<br>– Awareness campaigns against food waste and critical consumption<br>– Territorial regeneration interventions<br>– Facilitating for the weakest social groups, so that they can access the supply chain (issue of meal vouchers, for example)<br>– Campaigns to promote cultural biodiversity<br>– Protection protocols for workers, regarding	– The characterizing elements are not clusterable.

					contractual conditions<br>– Control systems of:<br>1. contractual equity<br>2. environmental compliance of production processes<br>– Projects concerning:<br>1. environmental regeneration<br>2. social aggregation<br>3. enhancement of sustainable local production.	
